# Atypical integration of temporal evidence and priors in causality judgment along the autism-schizotypy continuum

**DOI:** 10.1016/j.isci.2026.115325

**Published:** 2026-03-13

**Authors:** Gianluca Marsicano, Michele Deodato, David Melcher

**Affiliations:** 1 Psychology Program, Division of Science, New York University Abu Dhabi, Abu Dhabi, United Arab Emirates; 2 Center for Brain and Health, NYUAD Research Institute, New York University Abu Dhabi, Abu Dhabi, United Arab Emirates

**Keywords:** behavioral neuroscience, sensory neuroscience, cognitive neuroscience

## Abstract

Perceptual inference arises from integrating sensory evidence with prior knowledge. Causality perception—deciding whether one event causes another—offers a window into this process. We examined how prior experience and individual differences shape causal inference in 150 neurotypical individuals spanning the autism-schizotypy (ASD-SSD) spectrum. Participants judged causality in dynamic collision events with varying temporal delays. Causality judgments were influenced by physical timing (sensory driven) and serial dependence on previous decisions (prior driven). SSD-like individuals showed the strongest serial dependence and ASD-like individuals the weakest. Hierarchical drift diffusion modeling revealed increased causality bias and lower decision thresholds in SSD-like individuals, reflecting a prior-dominated style. ASD-like individuals showed reduced perceptual-history influence and higher thresholds, indicating a sensory-driven approach. Crucially, prior biases and perceptual history were interrelated, suggesting a hierarchical organization of perceptual inference across timescales and linking causality perception to predictive processes shaped by distinct cognitive-perceptual profiles.

## Highlights

• Sensory evidence and prior expectations jointly shape causality judgments
• Prior decisions exert serial dependence biases on current causality judgments
• SSD-like individuals show increased starting and perceptual causality biases
• ASD-like individuals are more sensory driven and show no causal biases

## Graphical abstract

**Figure:**
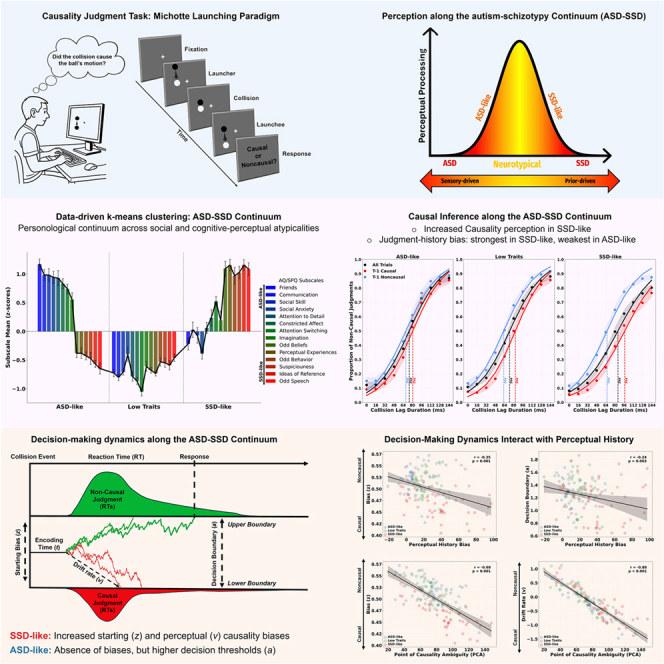

## Introduction

Human perception is not a passive reflection of sensory input but a dynamic inferential process through which the brain constructs coherent representations of the external world. A paradigmatic example is causality perception—the ability to perceive one event as the cause of another. This capacity is considered automatic and is present even in newborns,^1^^,^^2^^,^^3^ reflecting the brain’s predisposition to extract causal structure from sensory input. Importantly, the ability to perceive causality underpins core cognitive functions, shaping our understanding of physical and social events, informing judgments about personal agency, and influencing how we interpret events in terms of our beliefs in natural or supernatural forces.^4^

A classic illustration of causality perception is the “launching effect,”^5^ where a moving object collides with a stationary one, causing the latter to appear set into motion. Critically, the impression of causality depends on timing: it emerges reliably when the second object moves within a narrow temporal window after the collision.^5^^,^^6^ Even in the absence of actual physical contingency, sensory information can be integrated and experienced as causally related when presented within this temporal-binding interval, whose breadth shows substantial interindividual variability.^7^

Importantly, causality attribution extends beyond spatiotemporal cues, with individual differences in temporal binding thresholds reflecting the influence of top-down processes, including beliefs about physical principles,^2^ perceptual experience,^8^^,^^9^ and decision-making dynamics.^10^^,^^11^

The predictive processing framework provides a powerful account of how causality perception emerges from the brain’s inferential dynamics. It posits that the brain operates as a hierarchical Bayesian inference engine, continuously generating predictions and updating them in light of sensory input.^12^ In this framework, causality perception arises from the interplay between sensory evidence and top-down prior beliefs, with individuals varying along a continuum from sensory-driven to prior-driven processing styles.^13^^,^^14^

These opposing styles become particularly salient in the contrast between autism spectrum disorder (ASD) and schizophrenia spectrum disorder (SSD). These conditions can be conceptualized as opposite poles on a continuum of predictive precision.^14^^,^^15^^,^^16^^,^^17^^,^^18^ In ASD, perceptual inference is thought to rely heavily on sensory input and to underweight prior expectations.^19^ Conversely, in SSD, inference is often described as overly prior driven and characterized by rigid model updating.^16^ However, both hypo- and hyperprior states can coexist within the same individual, with aberrant perception arising from misallocated precision across different levels of the predictive hierarchy.^20^ Specifically, low-level sensory information may be underweighted, leading to excessive and noisy prediction errors. To impose coherence on this uncertainty, the system may overcompensate by assigning excessive precision to high-level beliefs, as observed in SSD.^20^ In line with this framework, core SSD features (e.g., delusions, ideas of reference, and anomalous perceptual experiences) have been linked to excessive reliance on internal models and widened temporal-binding windows.^21^^,^^22^^,^^23^ This imbalance fosters a stronger bias toward structured interpretations and faster evidence accumulation toward prior-consistent outcomes, manifesting as increased susceptibility to intentionality and causality attributions.^9^^,^^24^^,^^25^ In contrast, individuals with ASD typically exhibit sensory-driven perceptual styles, characterized by greater reliance on sensory evidence and reduced top-down prior influence.^19^^,^^26^^,^^27^ This style contributes to difficulties in integrating temporally distributed events into coherent causal narratives, as well as challenges with ambiguity resolution and contextual integration.^19^^,^^28^ Overall, these diverging perceptual styles may reflect distinct precision-weighting mechanisms operating across hierarchical levels of inference, giving rise to unique neurocognitive profiles in both clinical and non-clinical populations.^19^^,^^20^

Additionally, cause-effect interpretations depend on, and are continuously updated by, both recent perceptual experience and previous perceptual decisions.^9^^,^^29^ This phenomenon, known as *serial dependence*,^30^ reflects the tendency for current perceptual judgments to be biased toward prior experience (i.e., choice- and trial-history bias^29^^,^^31^), serving as an adaptive mechanism that promotes perceptual stability. Initially documented for low-level features, serial dependence also shapes higher-order processes such as causality perception. Previous findings showed that while prior stimulus experiences exert a strong influence on current causality judgments, interestingly, events following a causal impression are more likely to be perceived as causal, independently of the spatiotemporal properties of the prior stimulus.^8^^,^^9^ However, the strength of serial dependence on previous decisions varies across individuals. When overly strong, it may foster perceptual rigidity; when too weak, it may impair the adaptive updating of internal models.^29^^,^^32^ This opposing influence of serial dependence on choice history is evident along the ASD-SSD continuum: SSD traits are associated with stronger dependence on prior choices, reinforcing biased causal attributions, whereas ASD profiles are linked to a reduced influence of choice history, resulting in slower updating of internal representations.^32^^,^^33^^,^^34^^,^^35^

Taken together, these findings highlight that causality perception arises from the interplay of sensory processing, top-down mechanisms, and the dynamic updating of internal representations, components that are dynamically weighted and may interact differently across neurocognitive phenotypes.^16^^,^^20^^,^^21^^,^^28^ While these components are often investigated separately, they do not operate in isolation; rather, each stage of the perceptual inference process dynamically interacts with the others to shape the final perceptual outcome.

In the current study, we aimed to disentangle how sensory input and top-down components of perceptual inference interact to shape causality perception in the neurotypical population. Building on previous evidence, we hypothesized diverging predictive styles in causality inference, consistent with hierarchical models in which perception emerges from the precision-weighted integration of bottom-up sensory signals and top-down expectations.^20^ We applied this framework dimensionally, reasoning that individual differences in cognitive-perceptual traits may reflect variability in how perceptual precision is distributed across hierarchical levels of sensory evidence and prior expectations, influencing how causal events are perceived. Based on this rationale, we expected that individuals with SSD-like profiles, marked by cognitive-perceptual atypicalities such as delusions and hallucinations, would exhibit increased causality perception, reflecting a prior-dominated inference style in which serial dependence on previous causality judgments reinforces biased causal attributions.^9^^,^^16^^,^^36^ In contrast, individuals with ASD-like profiles were expected to rely more strongly on sensory evidence, resulting in slower model updating and a reduced influence of recent choice history.^28^^,^^32^^,^^34^^,^^37^^,^^38^ Finally, we expected individuals without prominent cognitive-perceptual atypicalities to exhibit a more balanced predictive style, flexibly integrating sensory input and prior models.

**Figure 1:**
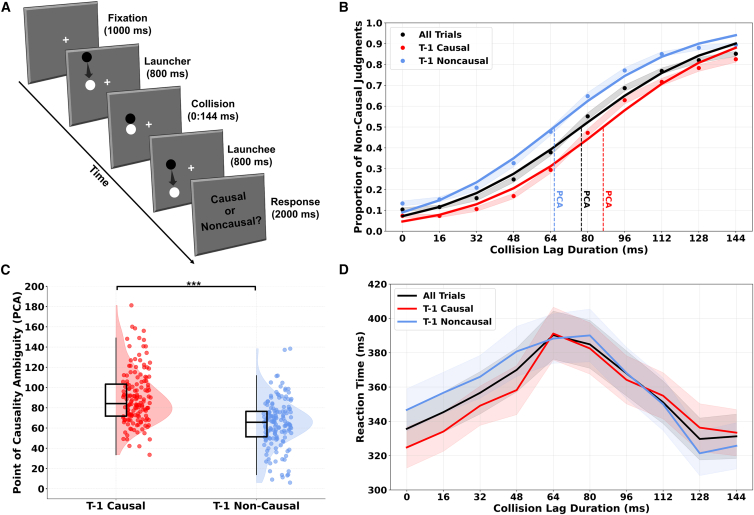
Experimental design and overall results across participants (A) Visual causality task design. Each trial began with a central fixation cross (1 s), followed by the appearance of two circles on one side of the screen. The first circle moved for 800 ms toward a stationary target at midline and collided with it. To manipulate causality perception, the onset of motion in the second circle was delayed by one of ten temporal lags (0–144 ms, in 17 ms steps). Participants were instructed to respond as soon as possible after the moment of collision and were allowed to respond immediately following this event. If no response was made during stimulus presentation, a response screen (“causal or non-causal?”) appeared after the motion sequence. (B) Psychometric functions and choice-history effects on causal perception. The figure depicts mean non-causal response rate across collision time lags for all participants. Psychometric functions are plotted for all trials (black), trials following a causal response (red), and trials following a non-causal response (blue). Vertical dotted lines represent the point of causality ambiguity (PCA; 50% threshold of the psychometric function) for each condition, operationalized as the delay at which participants were equally likely to report a causal or non-causal percept. Shaded areas indicate the standard error of the mean (SEM). (C) Choice-history effects on the PCA. Raincloud plot illustrating the difference in PCAs between trials preceded by a causal (blue) versus non-causal (red) judgment. PCAs were significantly higher following causal trials, indicating an influence of recent perceptual choice history on causal judgments (serial dependence effect). Black boxplots indicate the median and interquartile range (IQR), and individual dots represent participant-level data. (D) Temporal dynamics of causality perception. Mean reaction times (RTs) at each collision lag, shown separately for all trials (black), t-1 causal trials (red), and t-1 non-causal trials (blue). RTs peaked at intermediate lags, where causality judgments tend to be most ambiguous, and were shorter at clear-cut lag intervals. Shaded areas represent SEM.

To test these hypotheses, we used a *Michotte launching paradigm* (Figure 1), in which 150 neurotypical participants judged the perceived causality of collision events with varying temporal lags. We characterized phenotypic variability along the ASD-SSD continuum by applying a data-driven *k*-means clustering approach to self-reported perceptual, cognitive, socio-affective, and communicative traits, stratifying participants into three subgroups: ASD-like, SSD-like, and low-trait (LT) profiles. We then examined how these individual differences influenced causality inference, first by assessing temporal thresholds for causality perception and evaluating the extent to which prior decisions biased current causal judgments. Finally, we applied a computational hierarchical drift diffusion model (HDDM^39^) to decompose the perceptual decision-making dynamics underlying causality judgments. This modeling approach allowed us to examine, across groups, whether the starting point (i.e., starting bias) of the decision process was biased toward either causal or non-causal interpretations, reflecting internal expectations even before sensory evidence was processed. It also enabled us to assess whether the accumulation of evidence was biased toward one response option as information unfolded over time (i.e., drift rate), and how much evidence participants required before reaching a final decision (i.e., decision boundary), thereby revealing more cautious or more impulsive decision-making strategies. Together, this approach enabled us to disentangle the sensory, top-down, and choice history-dependent components of perceptual inference in causality perception.

## Results

### Cluster analysis: Individual profiles along the ASD-SSD continuum

To investigate how individual differences influence the construction of causality perception, we first characterized the multiple potential phenotypes observable in our sample (*n* = 150 neurotypical participants) computing a data-driven *k*-mean clustering approach on self-reported perceptual, cognitive, socio-affective, and communicative styles derived from the Autism Quotient (AQ^40^) and Schizotypal Personality Questionnaire (SPQ^41^). Silhouette scores of the clusters for values of *K* ranging from 1 to 10 were calculated to determine the optimal number of clusters (*K*), selecting the *K* producing the highest silhouette score. According to the silhouette coefficient (0.36), the optimal clustering solution was 3 *K*, suggesting a reasonable 3-cluster structure^42^ (see Table 1 for clustering summary).

**Table 1:** *k*-means clustering-derived solutions along the autism-schizotypy continuum

N Clusters	R^2^	BIC	Silhouette
2	0.290	1620.39	0.28
3	0.532	1143.29	0.36
4	0.520	1134.16	0.34
5	0.420	1187.39	0.31

Cluster solutions derived from the clustering analysis. The table presents adjusted coefficient of determination (R^2^), Bayesian information criterion (BIC), and Silhouette scores for the cluster solutions obtained. The analysis revealed higher R^2^ and Silhouette score for the 3-cluster structure stratifying participants in ASD-like traits, low traits, and SSD-like traits.

**Figure 2:**
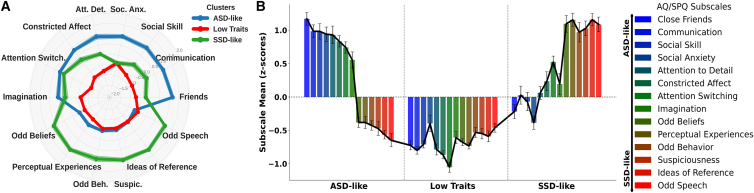
Data-driven *k*-mean cluster analysis: ASD-SSD like sub-phenotypes (A) Radar plot of subscale scores across clusters. Radar plots depict mean *Z* score values for each subscale of the Schizotypal Personality Questionnaire (SPQ) and Autism Spectrum Quotient (AQ) across the ASD-like, low traits, and SSD-like clusters. The ASD-like cluster is characterized by elevated socio-communicative-affective atypicalities (SPQ subscales: no close friends, social anxiety, constricted affect; AQ subscales: social skills, communication, attention to detail, attention switching, imagination). The SSD-like cluster shows prominent cognitive-perceptual anomalies (SPQ subscales: ideas of reference, odd beliefs/magical thinking, unusual perceptual experiences, odd behavior, odd speech, suspiciousness; AQ subscale: attention switching). The low traits cluster exhibits low scores across subscales. (B) Bar plot of mean subscale scores. Bar plots show the mean *Z* scores for each SPQ and AQ subscale across the three clusters. Error bars represent the standard error of the mean (SEM). Overall, the results highlight a diametric relationship between schizotypal and autistic traits, representing opposing poles of a continuum among individuals exhibiting atypicalities in cognitive-perceptual and socio-communicative-affective dimensions.

In line with previous evidence,^14^^,^^43^ the 3-cluster structure obtained in our analyses (Figure 2) comprised (1) a first cluster (*n* = 44, 29.3% of participants), named “ASD-like”, composed with individuals displaying high scores on the socio-affective dimension (SPQ subscales: *no close friends*, *social anxiety*, *constricted affect*; AQ subscales: *social skills*, *communication*, *attentional to detail*, *attention switching*, *imagination*) and low scores in the cognitive-perceptual domain (*SPQ*: *ideas of reference*, *odd beliefs*/*magical thinking*, *unusual perceptual experiences*, *odd behavior*, *odd speech*, *suspiciousness*); (2) a second cluster (*n* = 50, 33.3% of participants), called “SSD-like”, was characterized by individuals showing high scores on the cognitive-perceptual dimension (SPQ subscales: *ideas of reference*, *odd beliefs*/*magical thinking*, *unusual perceptual experiences odd behavior*, *odd speech*, *suspiciousness*; AQ subscales: *attentional switching*) and low scores in the socio-affective dimension (SPQ subscales: *no close friends*, *social anxiety*, *constriced affect*; AQ subscales: *social skills*, *communication*, *attention to detail*, *communication*, *imagination*); (3) a third cluster (*n* = 56, 37.3% of participants), called “low traits” (LT), composed of individuals with low scores in both AQ and SPQ subscales. The subscales *attentional switching* and *imagination* (AQ) showed comparable moderate scores both on ASD-like and SSD-like clusters and lower scores in LT cluster. Overall, these results align with previous findings highlighting a diametric relationship between autistic and schizotypal traits, representing opposing poles of a personological continuum among individuals exhibiting atypicalities in socio-communicative-affective and cognitive-perceptual dimensions.^14^

### Atypically increased causality inference in SSD-like profiles

We first examined whether judgments of causality in collision events during the *Michotte launching paradigm*^5^ (Figure 1) varied across different collision lags as a function of participant clusters identified through *k*-means cluster analysis, indexing each individual’s *point of causality ambiguity* (PCA) by fitting a logistic function to the proportion of non-causal responses as a function of collision time lags. The PCA corresponds to the 50% threshold of this function, which reflects the temporal delay at which participants were equally likely to report a causal or a non-causal percept. Within this framework, lower PCA values indicate a stronger tendency toward non-causal perception, whereas higher PCA values indicate a greater propensity to perceive causality. We performed a one-way ANOVA on PCA with cluster as between-subjects factor (three levels: ASD-like, LT, and SSD-like). The results revealed a significant main effect of cluster (F_(2,147)_ = 6.351, *p* = 0.002, η2*p* = 0.08), suggesting distinct profiles of causality perception within our sample of participants. SSD-like participants showed heightened PCA (M = 88.62, SD = 26.25), as compared to ASD-like (M = 74.23, SD = 22.8; *p* = 0.01, Cohen’s *d* = 0.589) and LT (M = 73.08, SD = 23.92; *p* = 0.004, Cohen’s *d* = 0.637) groups. On the contrary, no significant difference emerged from the comparison between ASD-like and LT (*p* = 0.814, Cohen’s *d* = −0.047). Overall, these findings offer insights into variability within the neurotypical population, consistent with previous evidence highlighting heightened causality perception in individuals showing prominent cognitive-perceptual atypicalities (e.g., delusional ideation, ideas of Streiling et al.^9^).

### Judgment-history bias: Attenuated in ASD-like, amplified in SSD-like participants

Subsequently, we investigated potential individual differences in the extent to which prior perceptual judgments influenced current causality perception by examining how preceding responses affected the current causality judgments across the different subgroups (i.e., *serial dependence*; judgment-history on trial *n*-1). Previous perceptual experience is typically integrated with current sensory information to support perceptual continuity over time.^30^ However, the effect of prior perceptual experience often shows substantial interindividual variability, particularly along the ASD-SSD spectrum. Individuals with high ASD traits tend to rely less on prior experience, favoring current sensory input,^37^ whereas individuals with SSD traits often exhibit a stronger reliance on stable prior models,^14^ reflecting distinct differences in how past information is integrated into current perceptual decisions.

**Figure 3:**
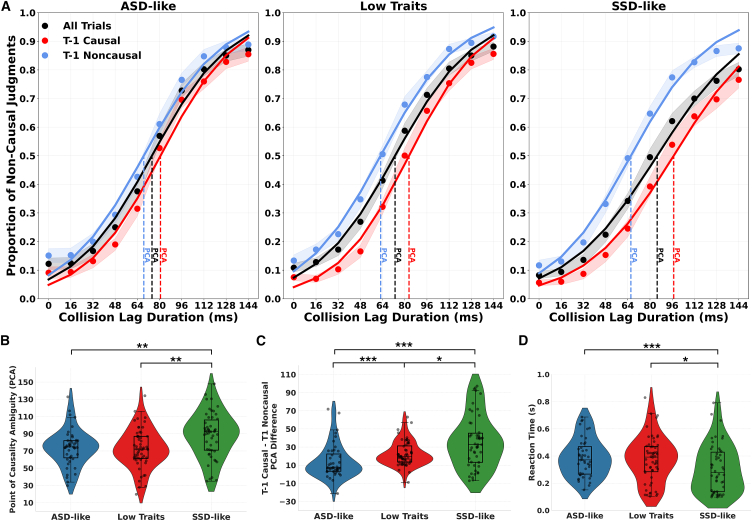
Group-level differences in causality inference (A) Psychometric functions of causal perception across groups. Psychometric functions showing the proportion of non-causal judgments as a function of collision time lag for each subgroup (left: ASD like; central: low traits; right: SSD like). Curves are plotted for all trials (black), trials following causal responses (red), and trials following non-causal responses (blue). Vertical dashed lines indicate the point of causality ambiguity (PCA; 50% threshold) for each condition. Shaded areas represent standard error of the mean (SEM). (B) Increased causality perception in SSD-like individuals. Violin plots of PCA values across subgroups. SSD-like participants showed significantly higher PCAs compared to both ASD-like and low traits groups, indicating a greater temporal tolerance for inferring causality and increased causality judgments. (C) Group differences in choice-history effects on causality perception. PCA difference scores (t-1 causal vs. t-1 non-causal) indexing serial dependence across subgroups. SSD-like participants exhibited significantly greater serial dependence effects than both ASD-like and low traits groups, suggesting a stronger influence of recent perceptual choice-history on causal judgments. (D) Divergent temporal dynamics underlying causality inference. Mean reaction times (RTs) across groups. SSD-like participants responded faster than ASD-like and low traits individuals, suggesting reduced decisional caution and a stronger prior-driven response style. In all plots, boxplots represent the median and interquartile range; violins depict the distribution; individual dots reflect participant-level data. Asterisks indicate significance levels (∗*p* < 0.05; ∗∗*p* < 0.01; ∗∗∗*p* < 0.001).

Accordingly, PCA was separately indexed for trials following causal (t-1 causal) and non-causal (t-1 non-causal) judgments. Since PCA distributions deviated from normality in two of the three clusters (ASD-like and LT), we performed a non-parametric 2 × 3 aligned rank transform (ART) ANOVA^44^ (see details in STAR Methods), with t-1 judgment (two levels: t-1 causal, t-1 non-causal) as the within-subjects factor and cluster (three levels: ASD like, LT, SSD like) as the between-subjects factor. The non-parametric Wilcoxon signed rank test was used to test differences within clusters and the Wilcoxon rank-sum test (Mann-Whitney U-test) to test differences across clusters (Holm-corrected). Consistent with previous findings, serial dependence significantly biased causality judgments (F_(1,147)_ = 199.91, *p* < 0.001, η2p = 0.576), with participants reporting more causal judgments following causal trials (M = 89.74, SD = 26.37) compared to non-causal judgments (M = 64.39, SD = 23.24; W = 11,049, *p* < 0.001, r = 0.83, large effect). Importantly, this analysis also revealed a significant main effect of cluster (F_(2,147)_ = 5.02, *p* = 0.008, η2p = 0.064) and a robust interaction between t-1 judgment and cluster (F_(2,147)_ = 11.77, *p* < 0.001, η2p = 0.138; Figure 3).

Within-group Wilcoxon signed-rank tests confirmed that prior perceptual judgment significantly influenced current causality judgments across all clusters, with an increased likelihood of causal responses following t-1 causality judgment relative to t-1 non-causality judgment trials (ASD like: t-1 causal M = 82.03, SD = 21.51 vs. t-1 non-causal M = 66.76, SD = 26.36, W = 918, *p* < 0.001, r = 0.63; LT: t-1 causal M = 84.32, SD = 19.94 vs. t-1 non-causal M = 61.77, SD = 23.14, W = 1593, *p* < 0.001, r = 0.72; SSD like: t-1 causal M = 102.58, SD = 31.74 vs. t-1 non-causal M = 65.32, SD = 20.46, W = 1247, *p* < 0.001, r = 0.79). Between-group Wilcoxon rank-sum tests further showed that, consistent with the notion of heightened causality perception in SSD, participants with higher SSD-like traits exhibited significantly higher PCA values following causal trials compared to both ASD-like (U = 652, *p* = 0.002, r = 0.39) and LT participants (U = 869, *p* = 0.002, r = 0.33), while no significant differences were found in the remaining comparisons (all *p* values ≥0.54).

Subsequently, to complement the aforementioned rmANOVA and to provide a clearer measure of the serial dependence effect (i.e., the extent to which prior perceptual judgment influenced current causality judgments) across clusters, we computed for each participant a PCA difference index (t-1 non-causal minus t-1 causal), with larger values indicating stronger bias from the previous perceptual judgment. We then compared the PCA difference index across clusters (between-subjects factor; three levels: ASD like, LT, SSD like) using a non-parametric Kruskal-Wallis test (since distributions violated normality). The analysis revealed a significant main effect of cluster (χ^2^(2) = 21.46, *p* < 0.001, η^2^[H] = 0.133, 95%; Figure 3C), indicating group differences in serial dependence strength. Post-hoc pairwise comparisons using the Dwass-Steele-Critchlow-Fligner (DSCF) procedure showed that SSD-like participants (M = 37.25, SD = 34.45) exhibited significantly higher PCA difference scores than both ASD-like participants (M = 15.36, SD = 24.16, *p* < 0.001, *r* = 0.39) and LT participants (M = 22.55, SD = 13.40, *p* = 0.042, *r* = 0.23). In addition, ASD like showed reduced serial dependence with respect to LT participants (*p* < 0.001, *r* = 0.37).

Overall, this pattern of results highlights distinct individual profiles in the weighting of prior perceptual judgment in causality judgments, with SSD-like individuals showing a stronger influence of previous perceptual experience, whereas ASD-like participants showed a reduced sensitivity to serial dependence on previous perceptual choices.

### Diverging reaction times in causality judgment along the ASD-SSD continuum

Next, we explored potential interindividual differences in the temporal dynamics of causal judgments by comparing reaction times (RTs) at each collision lag across clusters. Alongside accuracy, fast RTs can indicate efficient processing of cause-effect relationships, as atypicalities in response speed may reflect disruptions in the perception of temporal contiguity.^11^

Accordingly, since distributions deviated from normality in the SSD-like cluster, we first conducted a 10 × 3 non-parametric aligned rank transform ANOVA on RTs across all trials to assess whether the response speed of causality judgments was modulated by collision lag (within subject: 10 levels) and cluster (between subjects: ASD like, LT, SSD like). The results revealed a significant main effect of cluster (F(_2,147)_ = 4.06, *p* = 0.019, η2p = 0.045; Figure 3D), and Wilcoxon rank-sum tests (Holm-corrected) showed that SSD-like participants (M = 0.307 s, SD = 0.177 s) exhibited faster RTs compared to ASD-like (M = 0.388 s, SD = 0.14 s; U = 143,023, *p* < 0.001, r = 0.26) and LT participants (M = 0.375 s, SD = 0.16 s; U = 129,397, *p* = 0.172, r = 0.04), while the ASD like vs. LT between-group comparison was not statistically significant (*p* = 0.172). Interestingly, regardless of the between-subjects factor cluster, such analysis revealed a main effect of collision lag (F_(9,1323)_ = 15.08, *p* < 0.001, η2p = 0.082). Wilcoxon signed-rank comparisons across lags (Holm-corrected) further indicated that RTs were significantly slower at intermediate lags compared to shorter (e.g., Lag 62 vs. Lag 0: W = 2,353, *p* < 0.001, r = 0.51, large effect; Lag 78 vs. Lag 0: W = 2,598, *p* < 0.001, r = 0.47, moderate effect) and longer time lags (e.g., Lag 62 vs. Lag 128, W = 9,024, *p* < 0.001, r = 0.52, large effect; Lag 78 vs. Lag 144, W = 9,091, *p* < 0.001, r = 0.53), where the perception of causality tends to be more clear-cut, thus reflecting a nonlinear time course of RTs in causal causality judgments as a function of collision lag. However, this analysis did not reveal a significant interaction between cluster and time lag (F_(18,1323)_ = 1.48, *p* = 0.088, η2p = 0.015).

Subsequently, we conducted a non-parametric 10 × 2 × 3 ART ANOVA, with time lag (10 levels) and t-1 judgment (two levels: t-1 causal, t-1 non-causal) as within-subject factors, and cluster (three levels: ASD like, LT, and SSD like) as the between-subjects factor. Interestingly, the analysis revealed a significant interaction between time lag and t-1 judgment (F_(9,123)_ = 3.13, *p* < 0.001, η2p = 0.014), indicating that the influence of prior judgments varied across collision lags. Post-hoc Wilcoxon signed-rank tests (Holm-corrected) showed that RTs were significantly faster following t-1 causal relative to t-1 non-causal judgments at short collision lags, specifically at Lag 0 (t-1 causal M = 0.325 s, SD = 0.171 vs. t-1 non-causal M = 0.347 s, SD = 0.176; W = 4,209, *p* = 0.006, r = 0.22), Lag 16 (M = 0.334 s, SD = 0.177 vs. M = 0.357 s, SD = 0.184; W = 4,317, *p* = 0.012, r = 0.21), Lag 32 (M = 0.349 s, SD = 0.189 vs. M = 0.366 s, SD = 0.193; W = 4,526, *p* = 0.033, r = 0.17), and Lag 46 (M = 0.358 s, SD = 0.187 vs. M = 0.381 s, SD = 0.198; W = 4,083, *p* = 0.003, r = 0.24). These findings suggest that the history of judgments interacts with temporal features of collision events, reducing RTs for causality choices when they follow a prior causality judgment. For the remaining lags, no significant differences were observed between t-1 causal and t-1 non-causal conditions (all *p* values ≥ 0.138). Moreover, this analysis revealed no significant interactions involving cluster (all *p* values ≥0.304).

Overall, these findings suggest that individual profiles, collision timing, and judgment history significantly influence the temporal dynamics of causal perception. Specifically, SSD-like participants exhibited faster reaction times compared to ASD-like and LT groups, aligning with previous findings that individuals with elevated SSD-like traits often prioritize speed over accuracy.^10^^,^^25^ This pattern may reflect a cognitive-perceptual bias toward perceiving collisions as causal events, potentially driven by a more inflexible decision-making style that favors rapid judgments regardless of perceptual ambiguity.

### Modeling perceptual decision-making dynamics along the ASD-SSD continuum

Upon the observed differences across groups, we next computed HDDM^39^^,^^45^ to gain deeper insight into the latent cognitive mechanisms shaping causality judgments. HDDM provides a powerful Bayesian computational framework for decomposing choice and RTs data into core decision-making components, thereby revealing how individuals evaluate sensory evidence, integrate prior expectations, and translate these computations into perceptual decisions. When making a decision, individuals may not only rely on information gathered from the physical world but also incorporate prior knowledge and internal models to generate predictions, which can in turn bias perception and shape behavioral responses. According to the HDDM, perceptual decision-making between two alternatives can be described as a dynamic process in which, starting from an initial state that reflects prior expectations, sensory evidence is encoded and progressively accumulated over time until a decision threshold associated with one of the two choices is reached.^39^^,^^45^

**Figure 4:**
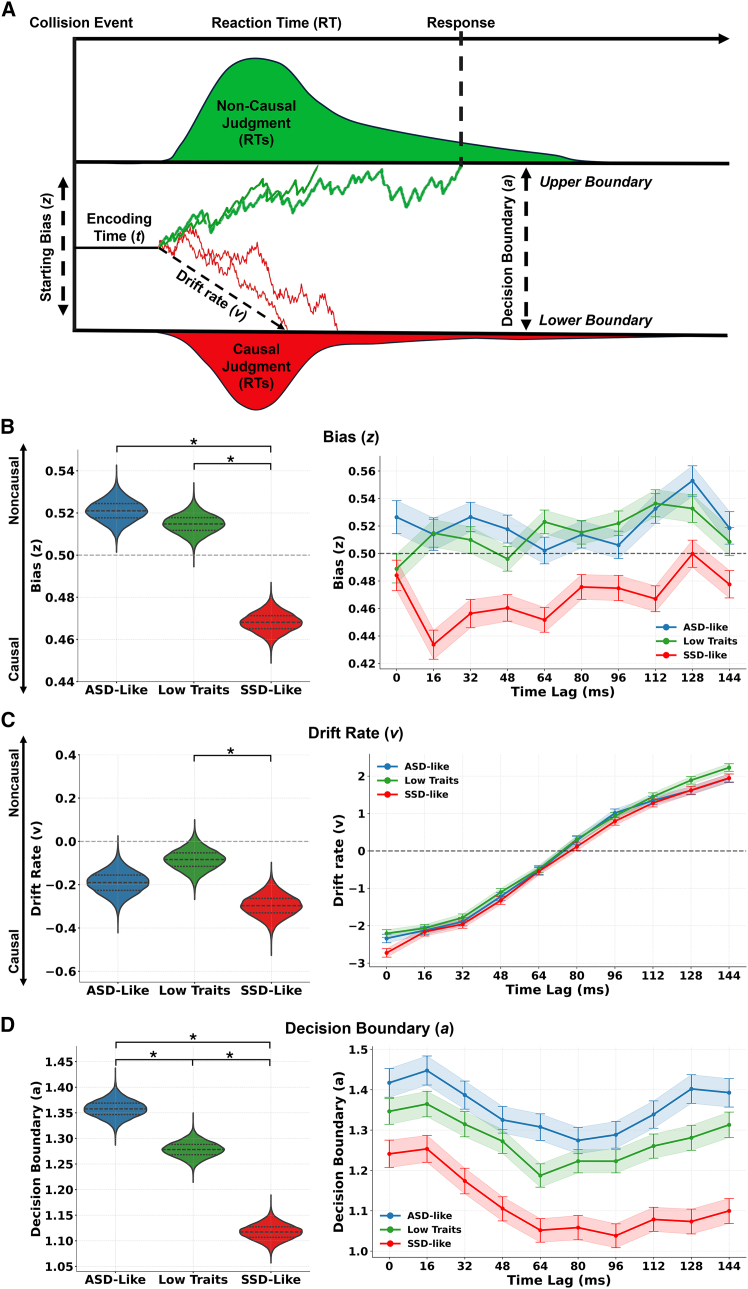
Hierarchical drift diffusion model: dissociable perceptual decision-making profiles along the ASD-SSD continuum (A) Hierarchical drift diffusion modeling (HDDM) of causality inference. Schematic depiction of the perceptual decision-making process captured by the HDDM implemented in this study. Following the collision between the moving objects (collision event), the non-decision time (encoding time, *t*) captures processes such as sensory encoding and motor execution that precede evidence accumulation toward a causal or non-causal judgment. Before evidence accumulation begins, the decision process starts from a starting bias (*z*), reflecting prior expectations that may favor either a causal or a non-causal outcome, or an unbiased starting point equidistant between the two decision alternatives. After this initial stage, sensory evidence is integrated over time at a drift rate (*v*), which quantifies both the efficiency and the direction of evidence accumulation. This parameter captures perceptual bias by indicating how strongly and in which direction sensory information is accumulated in favor of one alternative over the other, reflecting a bias toward causal or non-causal interpretations of the event when evidence is available. The decision process terminates when the accumulated evidence reaches one of two decision boundaries (*a*): in this case, the upper boundary corresponds to a non-causal judgment and the lower boundary to a causal judgment. (B) Increased starting bias toward causality in SSD-like individuals. Violin plots (left) and time course plots (right) of starting bias (*z*) toward causal responses across groups. The horizontal gray line at 0.5 indicates a neutral starting point, where the decision process begins equidistant between the two alternatives and is therefore unbiased toward either causal or non-causal judgments. Values above 0.5 reflect a starting bias toward non-causal decisions, whereas values below 0.5 indicate a bias toward causal decisions. SSD-like participants exhibited a significantly stronger initial bias toward causality compared to both low traits and ASD-like groups, consistent with a prior-driven inferential style. No credible difference emerged between ASD-like and low traits groups. (C) Greater perceptual bias in SSD-like individuals. Drift rate (*v*), reflecting the perceptual bias in the accumulation of sensory evidence toward a decision. Positive drift values indicate that evidence was accumulated more strongly in favor of non-causal judgments, whereas negative values indicate a perceptual bias toward causal judgments. SSD-like individuals showed a significantly higher drift rate for causal responses relative to low traits participants, indicating a stronger perceptual bias toward causal judgments. Drift dynamics across time lags (right) revealed a consistent pattern across groups: evidence accumulation was stronger at short lags (reflecting a strong causal impression), slowed near perceptual ambiguity (intermediate lags), and reversed at longer lags (favoring non-causal judgments). (D) Diverging decision thresholds in ASD-like and SSD-like groups. Decision boundary (*a*), indexing the amount of evidence required before making a decision. SSD-like participants showed lower decision thresholds compared to both ASD-like and low traits groups, suggesting a more impulsive decision policy. In contrast, ASD-like individuals exhibited the highest boundaries, reflecting greater decisional caution. Shaded areas and violin plot widths represent the 25^th^–75^th^ percentile range of the posterior distributions, with the central line indicating the median. Asterisks mark credible differences based on highest density intervals (HDIs).

In our study, we reasoned that individuals belonging to different clusters may differ in how they integrate prior knowledge with current temporal evidence, resulting in distinct perceptual decision-making dynamics underlying causality judgments. Specifically, we expected SSD-like individuals to exhibit a more biased evidence accumulation process, with the accumulation trajectory shifted toward the boundary associated with their initially favored option (i.e., the “causal” response). In contrast, ASD-like individuals were expected to show a more conservative decision-making strategy indicative of greater reliance on current sensory evidence. We hypothesized that such group differences could emerge through distinct underlying mechanisms that are well captured by the HDDM. These include a shift in the prior response preference (starting bias, *z*), which indicates a tendency toward one decision before evidence is accumulated. They may also involve a perceptual bias,^46^ reflected in a modulation of the uptake and direction of sensory evidence accumulation (drift rate, *v*), which captures differences in how strongly sensory information is accumulated in favor of one alternative. Additionally, they may result from variations in the amount of evidence required before committing to a decision (decision boundary, *a*), which is indicative of more cautious or more impulsive decision strategies (see Figure 4A for a graphical overview of the HDDM).

Accordingly, we fitted HDDM to the reaction time distributions associated with causality judgments. To this end, to identify the model that best captured the structure of our data, we tested 18 candidate models that embodied these predictions, each defined by different combinations of HDDM parameters that were allowed to vary as a function of collision time lags and participant clusters (see details in STAR Methods). In the baseline model (model 1), none of the four core HDDM parameters—encoding time (t), starting bias (z), drift rate (v), or decision boundary (a)—was allowed to vary as a function of collision time lag or participant cluster. Subsequent models were designed to systematically assess how each parameter might be influenced by these factors. In models 2–5, we tested whether each parameter was modulated by time lag alone, whereas models 6–9 examined whether they varied as a function of the participant cluster. Because the time lag between the moving dots directly affects the temporal dynamics of sensory evidence, with causal evidence typically available earlier and non-causal evidence emerging only after a delay, we further considered that model parameters might systematically depend on time lag. To address this, we first tested models in which z, v, and a varied as a function of time lag (model 10) or cluster (model 11), with t depending on time lag. We next tested models in which both time lag and cluster modulated specific subsets of parameters: drift rate and starting bias (model 12), drift rate and decision boundary (model 13), or starting bias and decision boundary (model 14), with encoding time depending on time lag. Finally, we tested three models in which starting bias, drift rate, and decision boundary were all modulated by both time lag and cluster, while encoding time was either fixed (model 15), allowed to vary with time lag (model 16), allowed to vary with cluster (model 17), or allowed to vary with both factors (model 18). Model fits were compared using the deviance information criterion (DIC), with lower values indicating better fit. The best-fitting model was the one in which starting bias, drift rate, and decision boundary depended on both time lag and cluster, and encoding time varied as a function of time lag (model 16, as indicated by the lowest DIC score). We performed several steps to validate this best-fitting model. These simulation-based analyses confirmed that the model accurately captured the core processes underlying perceptual decision-making in causality inference as observed in our data (see STAR Methods and Figures S2–S6).

Next, we characterized the posterior distribution of each parameter by indexing its median value and high-density interval (HDI), and compared posterior distributions by subtracting HDIs; if the HDI distribution of differences did not include zero, the condition effect was considered credible and thus statistically significant^47^ (see details in STAR Methods). First, to characterize individual differences in the latent decision mechanisms underlying causality inference, we examined group-level differences in the posterior distributions of the model parameters estimated at the group level (i.e., starting bias, drift rate, and decision boundary). Consistent with the hypothesis of heightened prior beliefs in cause-effect attribution, the SSD-like group exhibited a stronger initial bias (*z*) toward causality compared to both the LT group (HDI_difference_ = [0.058; 0.033]) and the ASD-like group (HDI_difference_ = [0.066; 0.039]; Figure 4V). Conversely, no credible differences emerged between the ASD-like and LT groups (HDI_difference_ = [-0.006; 0.019]; Figure 4B). Importantly, SSD-like individuals not only initiate decision-making with a stronger initial bias but also show a more strongly biased accumulation of sensory evidence toward causality (Figure 4C). Indeed, the SSD-like group showed a credible increased drift rate (v) toward causal responses with respect to LT participants (HDI_difference_ = [-0.346; −0.079]). On the other hand, no credible differences emerged from the comparison between the SSD-like and ASD-like groups (HDI_difference_ = [-0.249; 0.03]), nor between the ASD like and LT (HDI_difference_ = [-0.241; 0.031]). Additionally, decision thresholds (*a*) were reached at different times across groups (Figure 4D). SSD-like individuals made causality judgments more rapidly and with lower decision boundaries compared to both the LT (HDI_difference_ = [–0.201, −0.120]) and ASD-like groups (HDI_difference_ = [–0.283, −0.198]), whereas the ASD-like cluster showed higher decision boundaries with respect to LT participants (HDI_difference_ = [0.121, 0.037]), suggesting a more impulsive decision-making policy in SSD-like participants, and a more cautious response strategy in the ASD-like group.

Subsequently, we examined differences in the posterior distributions of the model parameters estimated at each collision time lag at the group level (i.e., starting bias, drift rate, and decision boundary). For encoding time (t), no group comparisons were performed, as this parameter was modeled solely as a function of time lag in the best-fitting model. Overall, the between-group credible differences previously identified remained consistent across time lags. Across all groups, evidence accumulation (drift rate) was faster toward causality responses at short time lags (negative *v*), approached zero at intermediate lags, reflecting the absence of biased evidence accumulation toward either response when perceptual ambiguity was highest and was faster toward non-causal judgments at long time lags (positive *v*; Figure 4C). Regarding the decision boundary, a different but complementary pattern emerged. Across groups, thresholds were higher at very short and very long time lags compared with intermediate lags, despite the fact that these conditions were associated with faster evidence accumulation. This may indicate that participants adopted a more cautious decision policy in situations where the sensory evidence was relatively unambiguous, suggesting that clearer evidence can prompt a more conservative decision criterion, possibly reflecting the need for stronger confirmation before endorsing a causal/non-causal interpretation.^45^^,^^48^

Additionally, an interesting pattern of results emerged from the comparisons within each cluster across the different time lags. The relation between collision time lag and decision boundary showed the previously described U-shaped pattern in the ASD-like and LT clusters, with thresholds being higher at short and long lags and lower at intermediate lags. In contrast, in the SSD-like cluster, thresholds were higher at short lags than at intermediate and long lags (Figure 4D).

Specifically, in the ASD-like cluster, decision boundaries were higher at lag 0 compared to lag 80 (HDI_difference_ = [–0.288, −0.0004]), and at lag 16 compared to lag 64 (HDI_difference_ = [–0.281, −0.0006]), 80 (HDI_difference_ = [–0.316, −0.035]), 96 (HDI_difference_ = [–0.299, −0.015]). In the LT cluster, thresholds were higher at lag 0 compared to lags 64 (HDI_difference_ = [–0.28, −0.03]) and lag 80 (HDI_difference_ = [–0.251, −0.0008]), at lag 16 compared to lags 64 (HDI_difference_ = [–0.302, −0.052]), 80 (HDI_difference_ = [–0.263, −0.014]), 96 (HDI_difference_ = [–0.269, −0.02]), and at lag 32 compared to lag 64 (HDI_difference_ = [–0.251, −0.006]). In contrast, the SSD-like cluster not only showed credible differences between short and intermediate time lags (e.g., lag 0 vs. lag 64: HDI_difference_ = [–0.325, −0.063]; lag 16 vs. lag 80: HDI_difference_ = [–0.325, −0.066]), but unlike the ASD-like and LT, also exhibited reliable differences between short and long time lags. Specifically, thresholds were higher at lag 0 compared to lag 96 (HDI_difference_ = [–0.33, −0.074]), 112 (HDI_difference_ = [–0.296, −0.035]), 128 (HDI_difference_ = [–0.298, −0.034]), 144 (HDI_difference_ = [–0.273, −0.01]). Similar differences were observed at lag 16 compared to lag 96 (HDI_difference_ = [–0.345, −0.09]), 112 (HDI_difference_ = [–0.301, −0.042]), 128 (HDI_difference_ = [–0.311, −0.048]), 144 (HDI_difference_ = [–0.289, −0.029]), and at lag 32 compared to lag 96 (HDI_difference_ = [–0.256, −0.006]). This distinctive pattern in the SSD-like cluster suggests that when sensory evidence increasingly supports a non-causal interpretation at longer time lags, decision thresholds are reduced compared to the other clusters, possibly reflecting greater confidence or a stronger prior bias that limits the adaptive adjustment of decision criteria to incoming evidence, consistent with previous accounts of inflexible evidence integration and altered predictive processing in SSD.^16^^,^^19^ Regarding non-decision time (*t*), which captures sensory encoding and execution processes and was modeled irrespective of cluster in the best-fitting model, we observed that *t* values were higher at very short time lags compared with several longer SOAs, whereas differences among intermediate and longer lags were not credible (posterior distribution of *t* across time lags: M = 0.134, SD = 0.053). In particular, *t* was greater at lag 0 compared to lags 16 (HDI_difference_ = [–0.028, −0.003]), 80 (HDI_difference_ = [–0.028, −0.003]), 112 (HDI_difference_ = [–0.026, −0.002]), 128 (HDI_difference_ = [–0.032, −0.008]), and 144 (HDI_difference_ = [–0.029, −0.005]), and credible differences were also found between lags 32 and 128 (HDI_difference_ = [–0.025, −0.002]). This pattern may suggest that perceptual encoding and response-related processes were prolonged when the two events occurred simultaneously, possibly reflecting increased demands for temporal integration and causal binding under conditions of maximal synchrony, or a delayed initiation of evidence accumulation driven by heightened prior expectations and decision confidence.^20^

Overall, the HDDM results revealed distinct decision-making profiles underlying causality judgments: SSD-like individuals exhibited a stronger initial bias toward causality, faster evidence accumulation, and lower decision boundaries, whereas ASD-like individuals tended toward a more cautious and conservative decision-making strategy.

### Decision-making dynamics interact with perceptual judgment history to shape causal inference

Finally, we performed Pearson correlation analyses to test whether individual differences in decision dynamics predict variability in PCA and serial dependence.

**Figure 5:**
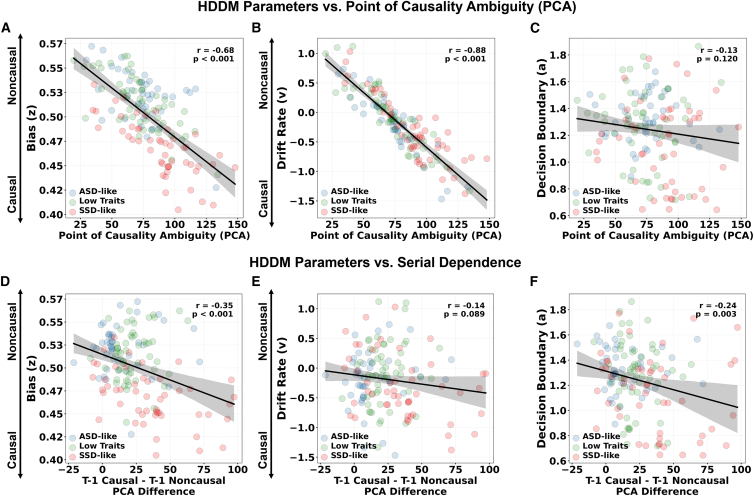
Causality Perception emerges from the interaction of perceptual priors and decision dynamics This figure shows scatterplots depicting Pearson correlations between hierarchical drift diffusion model (HDDM) parameters and the point of causality ambiguity (PCA), as well as between HDDM parameters and the serial dependence effect (defined as the difference in PCA following t-1 causal vs. t-1 non-causal responses). (A) Starting bias predicts causal perception. Starting bias (z) was strongly negatively associated with PCA (*r* = −0.68, *p* < 0.001). (B) Perceptual bias predicts the point of causality ambiguity. Drift rate (v) showed a strong negative association with PCA (*r* = −0.88, *p* < 0.001). These results indicate that individuals with a stronger initial bias and greater accumulation of evidence toward causal interpretations required longer temporal delays before shifting to non-causal interpretations. (C) Decision thresholds of causality judgments. No significant association was observed between PCA and decision boundary (a) (*r* = −0.12, *p* = 0.11). (D) Interaction between starting and dynamic biases. Greater serial dependence on previous choice was associated with lower starting bias values (*r* = −0.34, *p* < 0.001). (E) Evidence accumulation does not predict perceptual history dependence. No significant relationship was found between serial dependence and drift rate (*r* = −0.14, *p* = 0.08). (F) Decision threshold predicts the influence of previous choice effects. Greater serial dependence was associated with lower decision boundaries (*r* = −0.24, *p* = 0.002). Together, these findings indicate that stable internal priors (starting bias), dynamic perceptual updating (serial dependence), and decisional strategies (decision boundary) jointly shape causality perception, suggesting a hierarchical inferential process differently modulated across individual profiles along the ASD-SSD continuum. In each scatterplot, each point represents one participant (*n* = 150) and is color-coded by subgroup (ASD like, low traits, SSD like). Solid lines depict the best-fitting linear relationship computed across all participants. Shaded bands represent 95% confidence intervals. Pearson correlation coefficients (*r*) and corresponding two-tailed *p* values are reported.

Regarding PCA, it was negatively correlated with the starting bias (z; *r* = −0.68, *p* < 0.001; Figure 5A) and drift rate (v; *r* = −0.88, *p* < 0.001; Figure 5B) but not with the decision boundary (a; *r* = −0.12, *p* = 0.11; Figure 5A). This pattern of results suggests that stronger priors (i.e., starting bias) toward causal interpretations reduce sensitivity in detecting accurate cause-effect relationships in colliding visual stimuli, as reflected in higher PCA values. Additionally, the speed of evidence accumulation (i.e., drift rate) was faster for causal judgments in participants with lower PCAs and faster for non-causal judgments in those with higher PCAs, indicating that drift dynamics align with individual differences in causality perception.

On the other hand, a stronger serial dependence on previous perceptual judgment history (i.e., difference between t-1 causal vs. t-1 non-causal trials) was negatively correlated with the starting bias (*z*; *r* = −0.34, *p* < 0.001; Figure 5A) and decision boundary (*a*; *r* = −0.34, *p* = 0.002; Figure 5B) but not with drift rate (*v*; *r* = −0.14, *p* = 0.08; Figure 5A). First, these results highlight that serial dependence and initial bias, although reflecting different sources of influence and operating at different timescales, interact strongly in reinforcing models of causality attribution. Thus, it is conceivable that strong serial influence from prior trials may reinforce the initial bias by stabilizing causal interpretations across time through a perceptual feedback loop in which biased perceptual decisions become increasingly self-confirming. Furthermore, higher decision thresholds predicted reduced dependence on perceptual choice-history in detecting cause-effect relationships, suggesting that participants who rely more on sensory evidence on a trial-by-trial basis may adopt a more cautious decision-making strategy, requiring more time to reach a decision.

## Discussion

In the current study, we sought to characterize and interpret the marked individual differences that shape causality perception. Our findings support the emerging view that causality perception is not a fixed, bottom-up process, but rather a multi-stage hierarchical inferential process. Across all participants, judgments of causality were modulated by both the temporal structure of the stimulus,^5^^,^^11^ and by recent perceptual experience, which introduced systematic serial dependence into perceptual decisions, reflecting an attractive mechanism that stabilizes perceptual inference over time.^8^^,^^9^ Moreover, individual differences in these processes were strongly linked to variability in top-down predictive models.^12^^,^^14^^,^^16^ This pattern supports contemporary frameworks in which perceptual inference arises from the interaction of bottom-up sensory input, prior beliefs, and dynamic updating mechanisms.^8^^,^^9^^,^^14^^,^^20^ Critically, these inferential dynamics did not operate uniformly across participants. Rather, they reflected systematic trait-dependent variability in how priors and sensory evidence are weighted, updated, and integrated in coherent perceptual representations.^14^^,^^36^

### Characterizing individual profiles along the ASD-SSD continuum

A central question motivating our study was whether trait-dependent differences in cognition and perception influence how cause-effect attributions are constructed. To capture this interindividual variability, we adopted a data-driven clustering approach, applying *k*-means clustering to self-reported perceptual, cognitive, socio-affective, and communicative styles. This strategy allowed us to identify naturally occurring phenotypic profiles, rather than imposing arbitrary thresholds. We identified three clusters: participants with high autistic traits (ASD like), participants with high schizotypal traits (SSD like), and participants with low traits (LTs) in both dimensions.

Our clustering structure aligned with prior evidence suggesting a diametric relationship between ASD- and SSD-like traits in the general population, whereby socio-communicative atypicalities are often accompanied by intact cognitive-perceptual abilities, and cognitive-perceptual atypicalities may coexist with relatively preserved social-affective functioning.^14^^,^^15^^,^^17^^,^^18^^,^^22^

Importantly, these profiles are also thought to reflect opposing poles on a continuum of predictive precision,^16^^,^^17^ with ASD profiles exhibiting sensory-driven inference,^19^ and SSD profiles exhibiting over-weighted priors and inflexible model updating.^16^^,^^17^^,^^20^ Accordingly, our stratification thus provided an ideal framework to test whether these opposing inference styles would manifest in systematic differences in causality perception, perceptual decision dynamics, and judgment history-dependent updating.

### Perceiving cause in time: Divergent inference styles along the ASD-SSD continuum

First, our results showed that SSD-like participants exhibited heightened causality perception, as reflected in higher temporal integration thresholds (i.e., point of causality ambiguity) compared to both LT and ASD-like participants. This finding aligns with prior work linking cognitive-perceptual atypicalities (e.g., delusional ideation, ideas of reference) to increased cause-effect attribution even in the presence of strong sensory cues.^9^ This imbalance may result in perceptual experiences that are coherent but inaccurate, ranging from hallucinations and delusions in clinical populations to subtler misattributions in subclinical manifestations. Additionally, core SSD-like features have been consistently associated with widened temporal binding windows for visual events,^21^^,^^22^^,^^23^^,^^49^ contributing to distorted sensory processing. This imbalance may explain the stronger bias toward structured interpretations, manifesting as increased susceptibility to temporal binding and causality attributions.^9^^,^^23^ These findings are consistent with recent hierarchical models of perceptual inference, suggesting that such biases may stem from misallocated precision across levels of the perceptual hierarchy.^20^ Specifically, diminished weighting of low-level sensory inputs may result in increased prediction error, prompting the system to over-rely on high-level beliefs to impose perceptual coherence, ultimately driving rigid causal interpretations, even under strong sensory evidence.

Interestingly, individual profiles also significantly predicted the temporal dynamics of causal perception, suggesting that differences in perceptual inference extend beyond accuracy alone. SSD-like participants exhibited faster response times, potentially reflecting a rigid decision-making style in which strong priors drive rapid, confident judgments.^10^^,^^25^ Conversely, ASD-like and LT participants exhibited slower response times, indicating a more sensory-driven, cautious approach to perceptual decisions. Together, these findings suggest that predictive processing differences along the ASD-SSD spectrum influence not only what is perceived but also how perceptual decisions unfold over time.

### Priors and perceptual decision-making dynamics along the ASD-SSD continuum

Differences in causality perception across the ASD-SSD continuum emerged both behaviorally and computationally, as revealed by HDDM. Consistent with our hypotheses, the SSD-like group exhibited a stronger initial bias (*z*) toward causality compared to both the LT and ASD-like groups. This starting bias was accompanied by a stronger accumulation of evidence toward causal responses, suggesting that SSD-like individuals not only begin the decision process with heightened expectations of causality but also exhibit a greater perceptual bias toward causal interpretations when processing sensory evidence. Additionally, the SSD-like group demonstrated lower decision thresholds, indicating a reduced need for sensory evidence before committing to a decision, aligning with prior findings showing that individuals within the SSD spectrum often favor speed over deliberation and show diminished tolerance for perceptual ambiguity.^10^^,^^25^^,^^50^^,^^51^^,^^52^ Thus, our findings point to an overarching perceptual style in SSD-like individuals, characterized by stronger priors toward causality, wherein causal structure is readily inferred even when strong temporal cues suggest the opposite (i.e., a non-causal perception), consistent with predictive processing accounts of the psychosis spectrum.^13^^,^^16^^,^^20^ While alternative explanations such as differential sensitivity to sensory information or reduced temporal sensitivity in SSD-like individuals remain plausible, our findings suggest that a causality prior biases perception toward interpreting ambiguous object interactions as causal, as previously reported in similar causality paradigms^9^^,^ and in other low-level perceptual domains.^53^ This prior increases the posterior belief in a causal link even when bottom-up sensory evidence becomes less supportive, for example when temporal delays are introduced. As a result, SSD-like participants may be more tolerant of such delays because their stronger prior belief in causality continues to dominate when the sensory evidence becomes perceptually ambiguous.^9^ In contrast, ASD-like participants did not exhibit atypical prior biases or deviations in evidence accumulation, but exhibited higher decision thresholds as compared to LT and SSD-like participants, suggesting a more conservative decision policy, requiring greater sensory evidence before committing to a perceptual judgment. This computational profile aligns with sensory-driven inference models of ASD, in which increased sensory weighting and reduced prior influence promote greater deliberation and caution in perceptual decisions.^18^^,^^28^^,^^49^^,^^54^ Taken together, these computational findings strongly support the existence of distinct perceptual inference profiles underlying causality judgments, providing a characterization of distinct precision-weighting strategies across hierarchical levels. However, it is important to note that the current HDDM provides a trial-wise characterization of decision dynamics. Specifically, in the present study, sensory evidence may evolve non-linearly or asymmetrically over time within a trial, whereas the model assumes a single, constant drift rate for each collision delay. Consequently, the estimated parameters should be interpreted as effective summaries of how collision delays influence decisions overall, rather than as direct descriptions of moment-to-moment evidence accumulation or asymmetric within-trial dynamics (for a detailed discussion, see limitations of the study).

### Not just stable priors: Perceptual judgment-history as a window into predictive processing

Beyond stable priors, our findings show that causality judgments are also shaped by dynamic influences from recent perceptual experience (i.e., *serial dependence*). We observed robust serial dependence effects across all groups, whereby prior judgments (*trial n-1*) significantly biased current causality perception. Crucially, the influence of previous perceptual judgment varied systematically across profiles, with SSD-like individuals exhibiting the strongest serial dependence effect and ASD-like individuals the weakest. These findings align with previous evidence suggesting that individuals with high SSD-like atypicalities place greater weight on prior perceptual experiences, reinforcing biased causal interpretations through a rigid, prior-consistent inferential style.^9^^,^^14^^,^^36^ Additionally, this pattern aligns with hierarchical predictive coding models of SSDs, wherein the underweighting of low-level sensory inputs increases reliance on recent high-level predictions to stabilize perception, ultimately promoting perceptual rigidity.^20^ In contrast, the reduced susceptibility to serial dependence observed in ASD-like individuals may reflect an absent or slower updating of internal representations and a more sensory-driven perceptual style, as often reported in the autistic spectrum.^13^^,^^19^^,^^28^^,^^34^^,^^37^ Together, these findings suggest that differences in how priors are dynamically updated across hierarchical levels contribute to individual variability in perceptual inference, supporting models of predictive processing in which both stable priors and adaptive updating mechanisms shape perception over time.

### Multiscale biases and decision dynamics interact in shaping perceptual inference

Together, these findings provide converging evidence that causality perception reflects a perceptual inference process where priors and decision-making dynamics vary across individuals, but how do these components jointly shape perception? First, our correlational findings suggest that prior perceptual choice contributes to systematic shifts in the starting point of the decision process, supporting the idea that recent perceptual experience and stable internal priors may interact and converge within a unified inferential framework.^29^ Indeed, our results indicate that these mechanisms may not operate independently: participants with a greater initial bias toward causality exhibited stronger serial dependence. This relationship highlights how strong priors and heightened sensitivity to serial dependence may interact to reinforce one another, creating a feedback loop in which each causal judgment increases the likelihood of perceiving causality in subsequent events. This perceptual “stickiness” may in turn contribute to cognitive rigidity and distorted causal attribution, as observed in SSD.^9^^,^^14^^,^^36^

We also found that lower decision thresholds were associated with stronger serial dependence, suggesting that participants who commit to perceptual decisions more quickly may be more susceptible to the influence of recent perceptual judgments. On the other hand, individuals with higher decision thresholds, more typical of ASD-like profiles, showed reduced reliance on perceptual choice-history, consistent with a cautious, sensory-driven processing style requiring greater evidence to update internal representations.^19^^,^^28^^,^^34^^,^^37^

Furthermore, the point of causality ambiguity was negatively correlated with both starting bias and drift rate. This suggests that a stronger initial bias and a biased accumulation of evidence toward causality required longer temporal delays before shifting to non-causal interpretations of the visual events, reflecting a more rigid cognitive-perceptual style and potentially accounting for the higher visual temporal binding thresholds observed in SSD.^21^^,^^22^^,^^23^

Overall, our findings support the view that causality perception emerges from the dynamic interplay of predictive stability, perceptual adaptability, and hierarchical precision weighting, revealing systematic individual differences in how perceptual decisions are shaped across the general population.

### Limitations of the study

Causality perception serves as a fundamental mechanism for parsing the sensory world and structuring temporal experience.^3^ Understanding its variability thus provides key insight into the architecture of perceptual processing. Our findings reveal that causality perception arises from a hierarchical and dynamic interplay of sensory input, internal priors, and prior perceptual judgment, and that these processes vary systematically across trait-based profiles in the general population. Stratifying participants based on ASD- and SSD-like traits revealed behavioral and computational markers of prior- and sensory-driven perceptual styles, highlighting that balanced perceptual processing depends on the flexible integration of these components, a capacity that may be diminished in both SSD and ASD profiles.

A limitation of the present study concerns the interpretation of the drift diffusion modeling results in light of the time-varying structure of the stimulus. In the visual causality paradigm used here, although we assumed that a single, linear diffusion process provides an adequate approximation of the decision-making process, sensory evidence relevant for causal versus non-causal interpretations was not static within a trial. During the interval between the stopping of the first object and the onset of motion of the second object, the absence of motion may differentially support competing interpretations, and the direction and strength of evidence may change once motion resumes. Under these conditions, evidence accumulation may be non-constant over time and may affect causal and non-causal interpretations asymmetrically. The HDDM employed in this study does not explicitly model such moment-to-moment fluctuations in evidence. Instead, collision time lag was treated as an experimental condition that parametrically shapes decision behavior. In this framework, drift rate should be interpreted as an effective parameter summarizing how a given collision delay biases decisions overall, rather than as a description of how sensory evidence evolves continuously within a trial. Nonetheless, extensions of the diffusion framework have been proposed that explicitly incorporate time varying or asymmetric evidence accumulation, including diffusion models for conflict tasks with time-dependent features^55^ and diffusion-based models of interval timing in which drift rates adapt dynamically as a function of temporal expectations.^56^ While such approaches are well suited to capturing within-trial temporal dynamics, they introduce substantial additional complexity and are not straightforward to implement within a hierarchical modeling framework. At the same time, standard drift diffusion models have recently been applied to other temporal perception tasks involving systematic manipulations of stimulus onset asynchrony. For instance, in audiovisual simultaneity judgment tasks—where evidence similarly unfolds over time within a trial and comparable modeling assumptions are explicitly acknowledged—diffusion modeling has been shown to provide a useful framework for decomposing decision behavior in tasks with time-varying stimuli, when parameters are interpreted at an appropriate level of abstraction.^57^ Despite these limitations, the HDDM implemented here provided a good fit to the behavioral data and enabled a principled decomposition of performance into decision-making components across collision delays and individual profiles. While alternative modeling approaches may provide a more detailed account of within-trial evidence dynamics, the present approach was well suited to our primary goal of comparing how temporal structure and individual differences modulate decision-making at the level of trial-wise inference. Future work integrating decision modeling with interval and event timing may help unify causal inference and temporal perception within a single computational framework.

Additionally, our trait-based classification was primarily based on self-report measures; future studies incorporating clinical assessments, neuroimaging, and physiological biomarkers could refine and validate these profiles. Moreover, whether similar trait-dependent differences extend to other domains of perceptual inference (e.g., agency, motion prediction, or affective interpretation) remains an open question. Finally, our findings underscore the importance of multidimensional models of predictive processing that account for individual variability across both typical and atypical populations. Future research should further explore how stable priors, dynamic updating, and decision policies interact across sensory and cognitive domains, and how these interactions shape variability in perception, cognition, and behavior.

## Resource availability

### Lead contact

Further information and requests for resources and reagents should be directed to and will be fulfilled by the lead contact, Gianluca Marsicano (gm3598@nyu.edu).

### Materials availability

This study did not generate new unique reagents.

### Data and code availability

• The trial-by-trial behavioral and questionnaire data for all participants included in this study are publicly available at https://https://osf.io/6cmna/.io/6cmna/.
• All code used for the data analyses is openly available at https://https://osf.io/6cmna/.io/6cmna/. Additional information is detailed in key resources table.
• Any additional information required to reanalyze the data reported in this paper is available from the lead contact upon request.

## Acknowledgments

This work was supported by the 10.13039/100012025NYUAD 10.13039/501100025180Center for Brain and Health, funded by Tamkeen under NYU Abu Dhabi Research Institute grant CG012.

## Author contributions

Conceptualization, G.M., M.D., and D.M.; methodology G.M., M.D., and D.M.; formal analysis, G.M.; software, G.M. and M.D.; visualization, G.M.; funding acquisition, D.M.; writing – original draft preparation, G.M. and D.M.; writing – review and editing, G.M., M.D., and D.M.; supervision, D.M.

## Declaration of interests

The authors declare no competing interests.

## STAR★Methods

### Key resources table

**Table:** 

REAGENT or RESOURCE	SOURCE	IDENTIFIER
**Deposited data**		

Behavioral data	This paper	https://osf.io/6cmna/

**Software and algorithms**		

MATLAB (R2021b)	Mathworks, Natick, MA, USA	https://www.mathworks.com
JASP (version 0.17.2.1)	JASP Team^58^	https://jasp-stats.org/
HDDM (Hierarchical Drift Diffusion Model) toolbox	Wiecki et al.^39^	https://hddm.readthedocs.io/en/latest/
Python version 3	Python Software Foundation	https://www.python.org
PsychoPy	Peirce^55^	https://www.psychopy.org/
RStudio	Posit team (2025)^59^	https://posit.co/download/rstudio-desktop/
Custom Code	This paper	https://osf.io/6cmna/

### Experimental model and study participant details

#### Participants

A total of 163 volunteers were recruited from the online experiment platform *Prolific* (www.prolific.com). Participants presented normal or corrected-to-normal vision and hearing. Exclusion criteria were self-reported neurological and attention disorders, epilepsy, and photosensitivity. During data collection, the refresh rate of the monitor was recorded for each participant, ensuring a correct timing of stimuli presentation at the desired refresh rate (i.e., 60 Hz). Four participants were excluded from subsequent analyses since they performed the task using a monitor with different refresh rate. We excluded an additional nine participants whose behavioral performance exhibited an Adjusted R^2^ value <0.9 following logistic fitting (see data analysis). The final sample included 150 participants (72 females, mean age = 23.7 years, SD = 3.79). We emphasized the critical importance of sitting in a dimly lit and quiet room and keeping a viewing distance of ∼50 cm from the screen. All participants gave informed consent by checking the relevant boxes on a consent form *Prolific* webpage and received 9.75$ as compensation. The experiment was conducted in accordance with the Declaration of Helsinki and approved by the New York University Abu Dhabi Human Research Protection Program Internal Review Board (Protocol approval number: HRPP-2020-129).

### Method details

#### Stimuli and experimental design

The task was created with PsychoPy^60^ and administered via Pavlovia (https://pavlovia.org/), a web-based platform for the presentation of psychophysics experiments via common web browsers. Visual stimuli were created using Psychtoolbox on MATLAB 2019a (MathWorks, Inc), generating 60 Hz-videos of launching sequence at different time lags. All stimuli were presented on a 60-Hz monitor at a recommended viewing distance of 50 cm against a uniform gray background. Each trial commenced with a fixation cross at the center of the screen presented for 1000 ms, followed by the presentation of a launching sequence. This sequence consisted of a circle moving toward and “colliding with” a second circle, subsequently “causing” the second circle to move in the same direction. The first circle moved toward the second circle for 800 ms before colliding with it. Upon collision, the display remained static, and to manipulate participants’ perception of causality, we introduced a temporal lag at the moment of collision. Specifically, we introduced 10 equally spaced collision lags ranging from 0 to 144 ms, increasing in steps of one frame, to elicit either causal or non-causal impressions. After the collision time lag, the second circle moved away for 800 ms before disappearing. Participants were instructed to report their causal versus non-causal judgment as soon as possible after the moment of collision, defined as the time at which the first circle contacted the second circle. If no response was registered during stimulus presentation, a response screen (“Causal or Non-causal?”) appeared after the offset of the second circle’s motion and remained visible for 2 s as a reminder. The stimuli moved vertically either upward or downward on the screen and were consistently presented in the left visual hemifield, with the initial position of the bounced circle aligned with the fixation point. Each combination of sequence direction and collision lag was presented 20 times, resulting in a total of 400 randomized trials divided into four blocks. Prior to the experimental session, participants completed 40 practice trials to familiarize themselves with the task and stimuli. During practice, participants were instructed to respond as soon as possible based on their subjective perception of causality. The experimental task lasted approximately 30 min per participant. Participants were instructed to maintain fixation while attending to the lateralized stimuli and to indicate via key press whether they perceived the interaction between the moving circles as causal. Importantly, they were explicitly advised not to treat the task as a lag detection task but rather to focus on their subjective impression of causality induced by the collision. Additionally, autistic and schizotypal traits (see next sections) were assessed at the beginning of the experimental session, prior to the causality perception task. The questionnaires were completed without time constraints, and participants were informed that they could take breaks both during their completion and before starting the behavioral task. The entire experimental session typically lasted 50–60 min. All participants were naive to the experiment’s purpose.

#### Autistic traits

Autistic traits were assessed using the Autism Spectrum Quotient (AQ^40^), a self-report questionnaire designed to evaluate multiple aspects of cognitive style, behavioral and socio-communicative patterns, and sensory experiences. The AQ consists of 50 items distributed across five subscales, each comprising 10 items: (i) attention to detail, (ii) attention switching, (iii) imagination, (iv) communication, and (v) social skills. Participants rated each item on a 4-point Likert scale, ranging from “definitely agree” to “definitely disagree,” indicating the extent to which they endorsed each statement. Following the original scoring method,^40^ we computed a total AQ score by summing the individual subscale scores, with higher values reflecting greater levels of autistic traits.

#### Schizotypal traits

Schizotypal traits were assessed using the Schizotypal Personality Questionnaire (SPQ^41^), a self-report measure consisting of 74 items. These items are distributed across nine subscales: ideas of reference, magical thinking, social anxiety, unusual perceptual experiences, constricted affect, lack of close friends, odd behavior, odd speech, and suspiciousness. The subscales are further grouped into three main factors: cognitive-perceptual, interpersonal, and disorganization. Participants responded to each item using a binary format (“Yes” or “No”), indicating whether they endorsed the statement. Following the original scoring method,^41^ responses were coded as 0 for “No” and 1 for “Yes,” with higher scores reflecting greater schizotypal traits.

### Quantification and statistical analysis

#### Psychometric function of visual causality judgments

We fitted a psychometric logistic curve to the percentage of non-causal judgments as a function of collision time lags using MATLAB (version R2021b; The MathWorks Inc., Natick, MA, USA). We performed the fitting of the psychometric function separately for each participant (mean adjusted coefficient of determination (R^2^) = 0.956). The adjusted R^2^ of the fitted model was evaluated individually, and participants with poor logistic fitting (adjusted R^2^ < 0.9) were excluded from the statistical analysis (participants excluded *n* = 9; final sample of participants *n* = 150). We used a logistic equation and a nonlinear least squares method to fit the proportion of non-causal responses as a function of collision time lags (Figure 1B). The formula applied was as follows: *y = 1/(1 + exp(b × (t - x))).* In this equation, *x* represents the time lag, and *y* denotes the proportion of non-causal responses. The lower bound of y was set at 0, and the upper bound was set at 1. The only free parameters of the function were *b* (the slope of the function) and *t* (the 50% threshold), both of which were constrained to assume positive values above zero. Individual 50% threshold values were obtained by fitting the psychometric logistic curve. This threshold reflects the Point of Causality Ambiguity (PCA), defined as the temporal delay at which participants were equally likely to report a causal or a non-causal percept. Conceptually, this measure is directly analogous to the Point of Subjective Equality (PSE^41^) in conventional psychophysics, as it captures the point of maximal ambiguity between two categorical judgments (in this case, causal vs. non-causal). Within this framework, a lower PCA value reflects a greater tendency toward non-causal perception, whereas a higher PCA value reflects an increased tendency to perceive causality.

Second, to investigate the impact of choice-history effect on visual causality judgments, we implemented a serial dependence approach,^61^ where all trials were divided into two bins based on whether they followed a causal (i.e., t-1 causal) or non-causal (i.e., t-1 non-causal) response trial (Figure 1). To ensure the robustness of this approach, we first examined the distribution of trial numbers across collision time lags and t–1 judgment conditions. Descriptive statistics revealed that trial numbers were uniformly and comparably distributed across lags in both bins, and that all participants contributed sufficient trials to allow stable psychometric fitting. Specifically, for the t–1 causal bin, the number of trials per lag was (values are reported as mean (M) and standard deviation (SD) across participants): lag 0 ms, M = 21.20, SD = 5.42, min = 8; lag 16 ms, M = 20.71, SD = 5.44, min = 6; lag 32 ms, M = 20.48, SD = 5.66, min = 6; lag 48 ms, M = 20.52, SD = 5.34, min = 6; lag 64 ms, M = 21.23, SD = 5.37, min = 5; lag 80 ms, M = 21.13, SD = 5.32, min = 8; lag 96 ms, M = 21.00, SD = 5.59, min = 9; lag 112 ms, M = 21.30, SD = 5.45, min = 7; lag 128 ms, M = 21.64, SD = 5.67, min = 9; lag 144 ms, M = 21.12, SD = 5.77, min = 6. For the t–1 non-causal bin, the corresponding values were: lag 0 ms, M = 18.44, SD = 5.46, min = 4; lag 16 ms, M = 18.94, SD = 5.41, min = 5; lag 32 ms, M = 19.10, SD = 5.67, min = 5; lag 48 ms, M = 19.00, SD = 5.31, min = 6; lag 64 ms, M = 18.36, SD = 5.32, min = 6; lag 80 ms, M = 18.45, SD = 5.34, min = 5; lag 96 ms, M = 18.65, SD = 5.60, min = 4; lag 112 ms, M = 18.32, SD = 5.51, min = 5; lag 128 ms, M = 17.96, SD = 5.70, min = 3; lag 144 ms, M = 18.40, SD = 5.74, min = 5. We then performed additional logistic fitting of non-causal responses for t-1 causal (mean adjusted R^2^ = 0.923) and t-1 non-causal trials (mean adjusted R^2^ = 0.938), obtaining a PCA estimate for each bin and participant. The difference between the PCA of t-1 causal and t-1 non-causal trials served as a proxy measure of the influence of previous trials on current causality judgments.

Additionally, to further investigate variations in visual causality judgments across each time lag, we examined each participant’s mean reaction times (RTs; Figure 1D), separately indexing all trials, t-1 non-causal trials, and t-1 causal trials.

#### Individual differences along the ASD-SSD continuum: Cluster analysis

To characterize the multiple phenotypes observable within the ASD-SSD continuum, previous studies have employed factorial (e.g., Principal Component Analysis^14^) and clustering (e.g., k-means/k-median^42^^,^^43^^,^^49^) approaches on measures derived from the AQ and SPQ questionnaires. In the present study, we chose to implement a cluster analysis technique rather than a factorial approach, as the latter is based on a variable-centered methodology that tends to underestimate the interindividual variability within a sample.^49^ In contrast, cluster analysis is a person-centered approach that reveals commonalities between individuals, thus identifying group-specific relationships between variables that are often obscured in a factorial approach. Accordingly, our sample was stratified using a k-means cluster analysis using JASP (version 0.17.2.1^58^) (Hartigan-Wong method with squared Euclidean distance, and a maximum of 25 iterations to find the optimal clustering solution) based on participants’ z-scored ratings on the five subscales of the AQ (*social skills, attentional switching, attention to detail, communication, and imagination)* and the nine subscales of the SPQ (*ideas of reference, social anxiety, odd beliefs/magical thinking, unusual perceptual experiences, eccentric/odd behavior, no close friends, odd speech, constricted affect, and suspiciousness*). These data-driven approach characterized our sample into three distinct clusters of participants along the ASD-SSD continuum: (i) ASD-like traits participants, (ii) Low Traits participants, and (iii) SSD-like traits participants (see results section for more details; Figure 2).

#### Hierarchical drift diffusion model (HDDM)

After stratifying our sample into three distinct clusters along the ASD-SSD continuum, we implemented a hierarchical drift-diffusion model (HDDM) using Python 3 and the HDDM toolbox.^39^ This approach enables the embedding of drift-diffusion models (DDMs) within a hierarchical framework and estimates parameters using Bayesian methods. A key advantage of HDDM is its ability to estimate parameters at both the individual and condition levels simultaneously, based on variable group-level distributions.

When making decisions, individuals rely not only on sensory information derived from the external environment but also on prior knowledge and internal models to generate predictions. These predictions can, in turn, bias perception and shape subsequent behavioral responses. Within the HDDM framework, it is possible to capture different measures that reflect distinct stages of latent perceptual decision-making dynamics. Perceptual decision-making between two alternatives is conceptualized as a dynamic process in which sensory evidence is first encoded (encoding time, t) and then gradually accumulated over time toward one choice (drift rate, v), starting from an initial state that reflects prior expectations and response preference (starting bias, z), until a decision boundary (decision threshold, a) associated with one of the two choices is reached.^39^ Here, we aimed to test whether individuals from different clusters differ in how they integrate prior expectations and response preferences with sensory evidence during causality judgments. Specifically, we examined whether SSD-like individuals show a stronger bias toward their preferred response option, whereas ASD-like individuals adopt a more conservative strategy with greater reliance on sensory evidence. A response bias can be modeled at the level of the starting point (starting bias, *z*), which can be shifted toward the preferred choice (in this case, causal vs. non-causal). Additionally, the evidence accumulation process (drift rate, *v*) can be biased toward one choice, indicating selective uptake of sensory information in favor of one alternative over the other (i.e., perceptual bias toward causal vs. non-causal). Finally, the decision boundary (*a*) captures the amount of evidence required before committing to a decision, with higher thresholds indicating a more cautious decision-making strategy and lower thresholds reflecting faster decisions. Here, we first fitted different HDDM models to the RT distributions associated with causality judgments in order to identify the model that best captured the structure of our data. We tested 18 candidate models, each defined by different combinations of parameters that were allowed to vary as a function of collision time lag and participant cluster:

Model 1: No parameter (*t, z, v, a*) varied as a function of collision time lag or participant cluster.

Model 2: Encoding time (*t*) only depended on time lag.

Model 3: Starting bias (*z*) only depended on time lag.

Model 4: Drift rate (*v*) only depended on time lag.

Model 5: Decision boundary (*a*) only depended on time lag.

Model 6: Encoding time (*t*) only depended on cluster.

Model 7: Starting bias (*z*) only depended on cluster.

Model 8: Drift rate (*v*) only depended on cluster.

Model 9: Decision boundary (*a*) only depended on cluster.

Model 10: All four parameters (*t, z, v, a*) depended on time lag.

Model 11: Starting bias (*z*), drift rate (*v*), and decision boundary (*a*) depended on cluster, encoding time (*t*) depended on time lag.

Model 12: Starting bias (*z*) and drift rate (*v*) depended on both time lag and cluster, encoding time (*t*) depended on time lag.

Model 13: Drift rate (*v*) and decision boundary (*a*) depended on both time lag and cluster, encoding time (*t*) depended on time lag.

Model 14: Starting bias (*z*) and decision boundary (*a*) depended on both time lag and cluster, encoding time (*t*) depended on time lag.

Model 15: Starting bias (*z*), drift rate (*v*), and decision boundary (*a*) depended on both time lag and cluster, encoding time (*t*) fixed.

Model 16: Starting bias (*z*), drift rate (*v*), and decision boundary (*a*) depended on both time lag and cluster, encoding time (*t*) depended on time lag.

Model 17: Starting bias (*z*), drift rate (*v*), and decision boundary (*a*) depended on both time lag and cluster, encoding time (*t*) depended on cluster.

Model 18: Starting bias (*z*), drift rate (*v*), and decision boundary (*a*) depended on both time lag and cluster, encoding time (*t*) depended on both factors.

All HDDMs were estimated using Markov Chain Monte Carlo (MCMC) sampling with four independent chains (10,000 iterations; burn-in = 5,000), resulting in 5,000 posterior samples per chain (20,000 in total) to approximate the posterior distributions of each parameter at both the individual and group levels. The prior distributions for each parameter were informed by a pool of 23 studies reporting best-fitting DDM parameters from previous perceptual decision-making tasks.^62^ Using empirically informed priors constrains parameter estimates to a plausible range based on prior literature, helps reduce issues with parameter collinearity, and improves the recovery of true parameter values^62^ (see also the supplement by^39^ for visual representations of these priors). Model fits were assessed by comparing Deviance Information Criterion (DIC) values, with lower values indicating a better fit, and by examining the Gelman–Rubin convergence diagnostic ($\hat{R}$), which evaluates the convergence of MCMC across multiple runs. Values of $\hat{R}$ close to 1.0 (typically <1.01) indicate good convergence and reliable parameter estimation.^62^ Detailed model fit comparisons (Table S1) and diagnostic assessments (Figures S1–S9) are reported in the supplemental information (see also below).

#### HDDM analysis and validation

Model comparisons showed that the best-fitting model was the one in which starting bias (*z*), drift rate (*v*), and decision boundary (*a*) depended on both time lag and cluster, while encoding time (*t*) varied as a function of time lag (model 16, DIC = 9174; see Table S1 for DIC and $\hat{R}$ model comparison).

We performed several steps to validate this best-fitting model. First, convergence of the HDDM posterior samples was assessed by inspecting the MCMC trace plots and their autocorrelation to ensure that the model had properly converged. Posterior distributions and MCMC trace diagnostics for all estimated HDDM parameters are reported in Figures S1 and S2, showing well-behaved, unimodal posteriors and stable chain convergence. Additionally, all parameters exhibited $\hat{R}$ values below 1.1, indicating optimal convergence of the MCMC chains.^62^

To further evaluate the goodness of the best-fitting model, we performed posterior predictive checks (PPCs) to assess how well it reproduced key aspects of the observed data. Using the posterior distributions of the model parameters, we generated 100 full simulated datasets replicating both response times and choices for each trial and participant. These simulations allowed us to evaluate whether the model could recover the empirical patterns of behavior beyond its relative fit indices. We then examined how well the model captured the reaction time distributions across groups (ASD-like, Low Traits, SSD-like; Figure S3), within each group across collision time lags (Figure S4), and at the individual subject level (Figure S5). The close overlap between simulated and empirical reaction time distributions provided strong evidence that the best-fitting model accurately captured the decision processes underlying participants’ behavior.

Additionally, to verify that the observed effects of cluster and time lag on the estimated parameters reflected genuine individual differences in perceptual decision-making rather than artifacts of the parameter estimation procedure, we conducted a parameter recovery analysis. Specifically, we generated a synthetic dataset by sampling subject-level parameters from the posterior distributions of the best-fitting model and then re-fitted this dataset using the same modeling approach described above for the best-fitting model. Recovery performance was assessed by correlating the generating parameters with those re-estimated from the simulated data, revealing strong and significant correlations for all parameters (*p* < 0.01; Figures S6 and S7), thus confirming the robustness and identifiability of the model estimates.

Finally, to further evaluate the robustness and identifiability of the model parameters, we conducted an additional analysis assessing the influence of priors specification on parameter estimation. Specifically, we re-estimated the best-fitting model using non-informative priors, which impose minimal constraints on the parameter space and allow posterior estimates to be driven primarily by the data, instead of the informative priors used in the main analyses. Informative priors, which are recommended in HDDM because they constrain parameter estimates to plausible ranges based on previous literature^62^^,^ and thereby help reduce parameter collinearity and improve recovery accuracy,^39^ were originally applied to guide estimation. In contrast, the non-informative priors provide a complementary check on the stability of the results by minimizing prior assumptions. We then correlated the parameter estimates obtained under the two prior specifications and found strong, significant correlations for all parameters (*p* < 0.001; Figures S8 and S9), indicating that our results were robust to prior assumptions and that the estimated parameters were reliably identifiable.

#### Descriptive statistics and assumption checks

Prior to hypothesis testing, we assessed distributional normality for all variables of interest within each cluster (ASD-like, Low Traits, SSD-like) using skewness, kurtosis, and the Shapiro–Wilk test, implemented in JASP (version 0.17.2.1^58^). For PCA, distributions were approximately normal in all clusters (ASD-like: skew = 0.320, kurtosis = 0.894, Shapiro–Wilk = 0.962, Shapiro–Wilk *p* = 0.158; Low Traits: skew = 0.069, kurtosis = 0.408, Shapiro–Wilk = 0.985, Shapiro–Wilk *p* = 0.727; SSD-like: skew = −0.147, kurtosis = −0.169, Shapiro–Wilk = 0.98, Shapiro–Wilk *p* = 0.534). For PCA by choice-history bin (i.e., serial dependence measures), t–1 causal trials showed modest positive skew in ASD-like and Low Traits (ASD-like: skew = 1.136, kurtosis = 2.734, Shapiro–Wilk = 0.928, Shapiro–Wilk *p* = 0.009; Low Traits: skew = 0.925, kurtosis = 1.894, Shapiro–Wilk = 0.953, Shapiro–Wilk *p* = 0.028), whereas SSD-like were approximately normal (skew = 0.010, kurtosis = −0.599, Shapiro–Wilk = 0.986, Shapiro–Wilk *p* = 0.807). For t–1 non-causal trials, all clusters were approximately normal (ASD-like: skew = −0.207, kurtosis = 0.335, Shapiro–Wilk = 0.976, Shapiro–Wilk *p* = 0.498; Low Traits: skew = 0.030, kurtosis = 0.648, Shapiro–Wilk = 0.977, Shapiro–Wilk *p* = 0.367; SSD-like: skew = −0.553, kurtosis = 0.567, Shapiro–Wilk = 0.971, Shapiro–Wilk *p* = 0.971). The PCA difference index (t–1 non-causal minus t–1 causal) exhibited positive skew in all clusters (ASD-like: skew = 1.402, kurtosis = 2.256, Shapiro–Wilk = 0.86, Shapiro–Wilk *p* < 0.001; Low Traits: skew = 0.816, kurtosis = 1.115, Shapiro–Wilk = 0.939, Shapiro–Wilk *p* = 0.007; SSD-like: skew = 0.570, kurtosis = −0.396, Shapiro–Wilk = 0.94, Shapiro–Wilk *p* = 0.014). For RTs, ASD-like and Low Traits were approximately normal (ASD-like: skew = 0.479, kurtosis = −0.507, Shapiro–Wilk = 0.96, Shapiro–Wilk *p* = 0.126; Low Traits: skew = 0.274, kurtosis = −0.008, Shapiro–Wilk = 0.965, Shapiro–Wilk *p* = 0.103), whereas SSD-like deviated (skew = 0.717, kurtosis = −0.066, Shapiro–Wilk = 0.923, Shapiro–Wilk *p* = 0.003). For RTs by choice-history bin, ASD-like and Low Traits were approximately normal (all Shapiro–Wilk *p*-values ≥0.08), while SSD-like deviated for both t–1 causal and t–1 non-causal (both Shapiro–Wilk *p*-values = 0.002). Based on these diagnostics, for statistical hypothesis testing we applied parametric models when normality assumptions were satisfied, and non-parametric approaches when violations were detected, ensuring that all findings were robust to distributional properties.

#### Statistical analysis

First, since distributions were normal in all clusters, we performed in JASP (version 0.17.2.1^58^) a one-way ANOVA on the PCA to test whether visual causality judgments were influenced by cluster (between-subjects factor with three levels: ASD-like, Low Traits, SSD-like).

To examine choice-history effects (i.e., serial dependence; trial *n*–1), we compared PCAs for trials following causal (t–1 causal) vs. non-causal (t–1 non-causal) responses across clusters. Because distributions in the t–1 causal condition deviated from normality in two of the three clusters (ASD-like and Low Traits), we employed a non-parametric 2 × 3 Aligned Rank Transform (ART) ANOVA^44^ - a rank-based approach that enables the analysis of factorial designs with interaction terms - with t–1 judgment (two levels: t–1 causal, t–1 non-causal) as a within-subjects factor and cluster (three levels: ASD-like, Low Traits, SSD-like) as a between-subjects factor (implemented in RStudio^59^). Post-hoc comparisons were conducted using Wilcoxon signed-rank tests for within-cluster effects and Wilcoxon rank-sum tests (Mann-Whitney U-test) for between-cluster effects, with Holm correction for multiple testing. Effect sizes for Wilcoxon tests were reported as rank-biserial correlations (r).

Additionally, to provide a direct summary measure of the serial dependence effect, we also computed for each participant a PCA difference index (t–1 non-causal minus t–1 causal), with larger values reflecting stronger dependence on the previous perceptual judgment. This single-value measure serves as a proxy of serial dependence strength, facilitating clearer visualization of between-group differences (e.g., Figure 3C) and providing a straightforward metric for judgment-history effect estimation. To complement the factorial rmANOVA described above, and to make group-level differences more interpretable, we statistically compared this index across clusters (between-subjects factor; three levels: ASD-like, Low Traits, SSD-like) using a non-parametric Kruskal–Wallis test, followed by Dwass–Steele–Critchlow–Fligner (DSCF) post-hoc comparisons, given that the variable of interest violated normality assumptions in all clusters (implemented in RStudio^59^). For RTs, since distributions deviated from normality in SSD-like cluster, we analyzed mean response times across time lags using a 10 × 3 non-parametric ART ANOVA, with time lag (ten levels) as the within-subject factor and cluster (three levels: ASD-like, Low Traits, SSD-like) as the between-subjects factor. To further assess serial dependence effects (i.e., judgment-history effects) on RTs, which also showed violations of normality, we conducted a 10 × 2×3 non-parametric ART ANOVA with time lag (ten levels) and t–1 judgment (two levels: t–1 causal, t–1 non-causal) as within-subject factors, and cluster (three levels: ASD-like, Low Traits, SSD-like) as the between-subjects factor (implemented in RStudio^59^). Post-hoc comparisons were conducted using Wilcoxon signed-rank tests for within-cluster effects and Wilcoxon rank-sum tests for between-cluster effects, with Holm correction for multiple testing. The Greenhouse–Geisser correction was applied where necessary to account for violations of the sphericity assumption. All post-hoc *p*-values were Holm-corrected.

Regarding HDDM, we characterized the posterior distribution of each parameter (i.e., Bias, Drift Rate, Decision Boundary) using its median value and high-density interval (HDI), separately for each cluster. For statistical inference (implemented in Python 3 and the HDDM toolbox^39^), we assessed between-groups differences by comparing posterior distributions by subtracting their HDIs. Specifically, for each contrast of interest, we computed differences based on the 20,000 iterations of each parameter estimation, and the resulting significance threshold was used to determine the boundaries of the high-density interval (HDI) distribution. If the HDI distribution of mean differences did not include zero, the condition effect was considered credible and thus statistically significant.^47^ Finally, we performed Pearson correlation analyses to test whether individual differences in the estimated HDDM decision-making parameters predicted variability in PCA scores and serial dependence effects.

Statistical analyses were conducted using JASP (version 0.17.2.1^58^), Python 3, MATLAB (version R2021b; The MathWorks Inc., Natick, MA, USA) and RStudio.^59^
