# Supplementary material for: Atypical integration of temporal evidence and priors in causality judgment along the autism-schizotypy continuum
Source: iScience. 2026 Mar 13;29(4):115325. doi: 10.1016/j.isci.2026.115325 (PMC13059111; doi:10.1016/j.isci.2026.115325)
Supplement: Document S1. Figures S1–S9 and Table S1 [file mmc1.pdf]

iScience, Volume *29*

Supplemental information

Atypical integration of temporal evidence and priors in causality judgment along the autism-schizotypy continuum

Gianluca Marsicano, Michele Deodato, and David Melcher

Supplemental Information

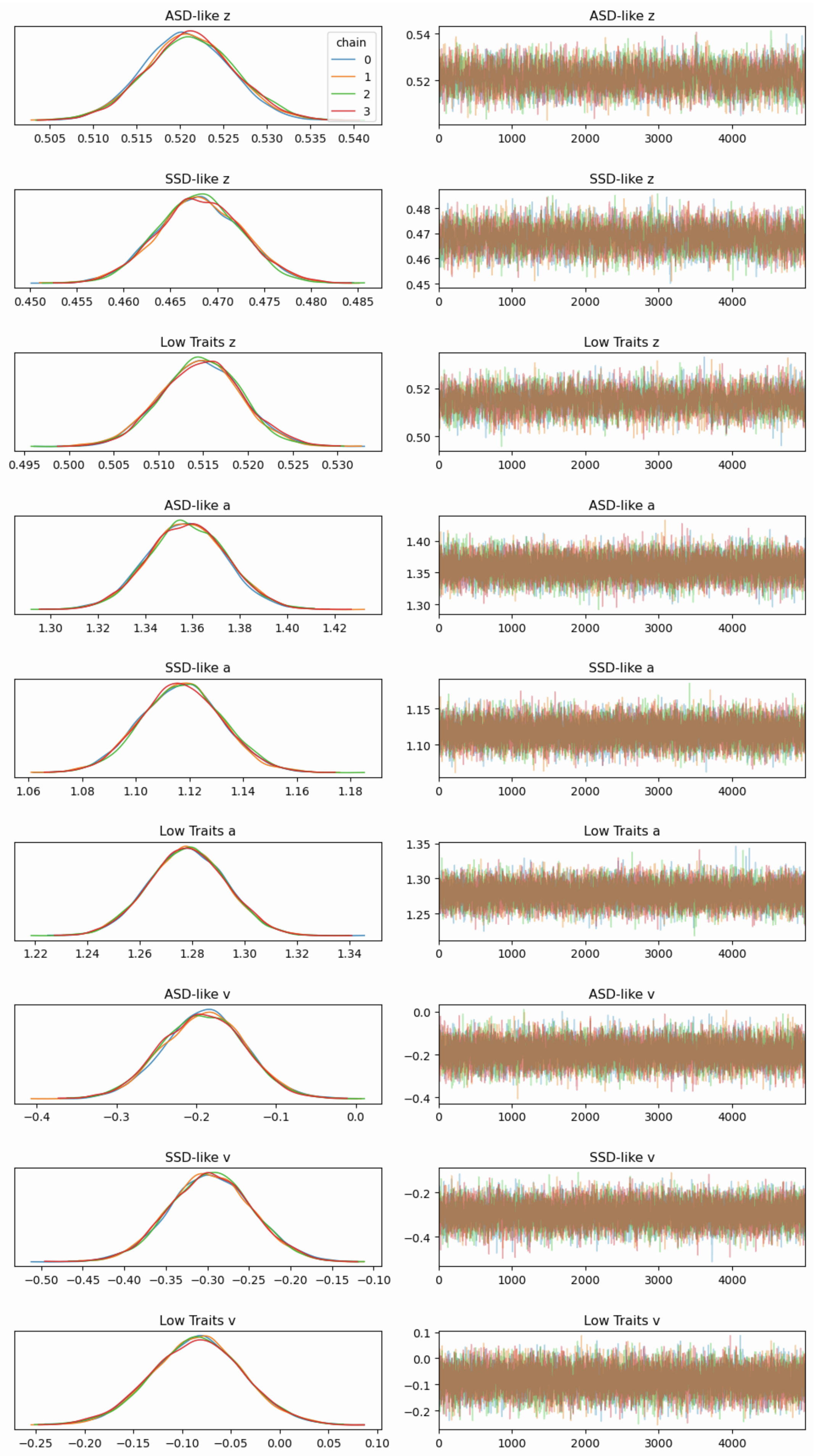
ASD-like z
chain
0
1
2
3
SSD-like z
Low Traits z
ASD-like a
SSD-like a
Low Traits a
ASD-like v
SSD-like v
Low Traits v

Figure S1. Traceplots and Posterior Distributions for each HDDM parameter across Clusters. Posterior distributions (left column) and Markov Chain Monte Carlo (MCMC) trace plots (right column) for the key decision-making parameters estimated via the Hierarchical Drift Diffusion Model (HDDM), shown separately for each group: ASD-like, SSD-like, and Low Traits. The posterior distributions (left) display well-formed, unimodal, and symmetric shapes, suggesting stable and reliable parameter estimation across groups. The trace plots (right) show the evolution of posterior samples across MCMC iterations, with consistent mixing and no visible drift, indicating good convergence of the model chains.

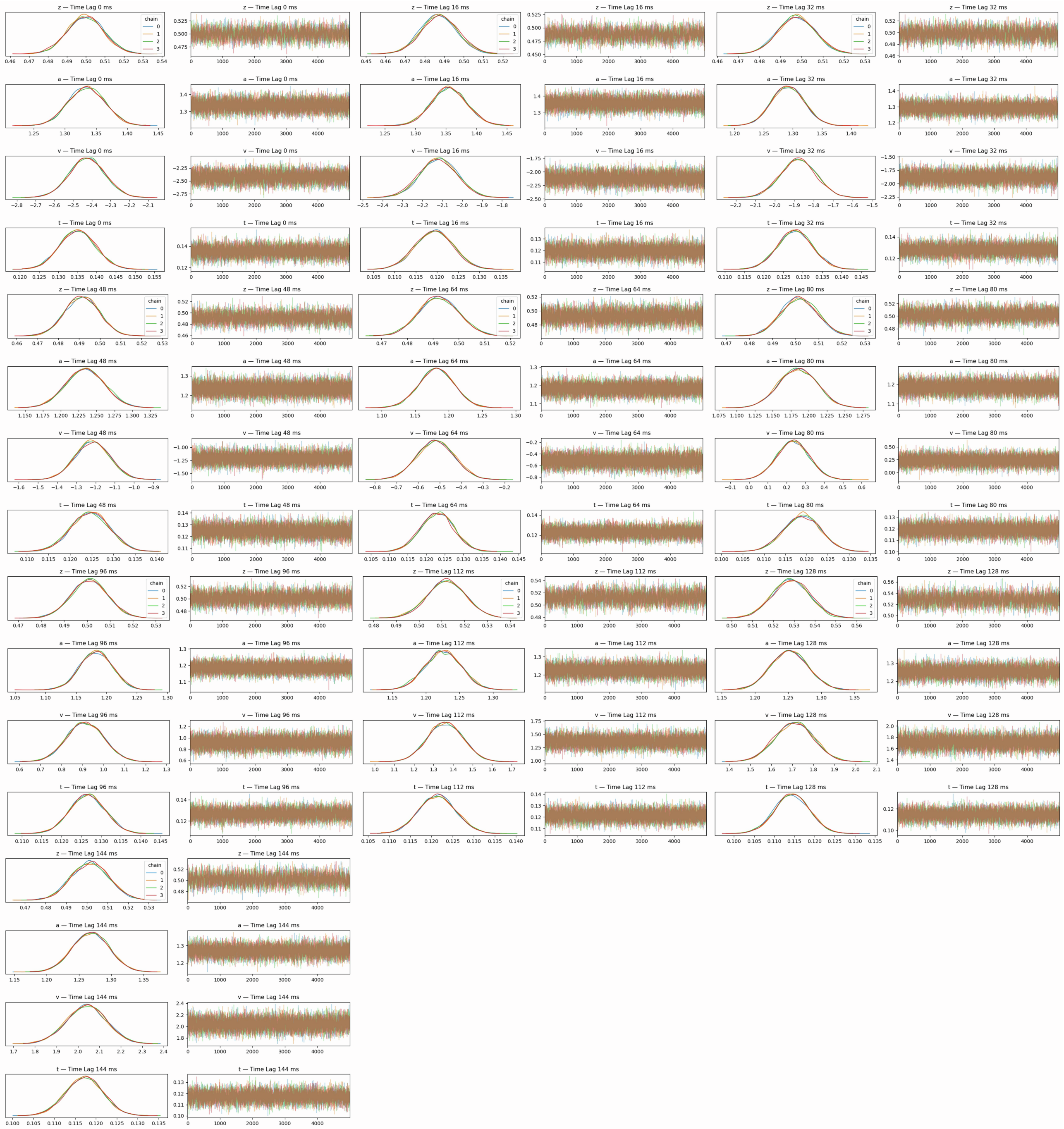

Figure S2. Traceplots and Posterior Distributions for each HDDM parameter across Collision Time Lags. Posterior distributions (left column) and Markov Chain Monte Carlo (MCMC) trace plots (right column) for the key decision-making parameters estimated via the Hierarchical Drift Diffusion Model (HDDM), shown separately for each time lag. The posterior distributions (left) display well-formed, unimodal, and symmetric

shapes, suggesting stable and reliable parameter estimation. The trace plots (right) show the evolution of posterior samples across MCMC iterations, with consistent mixing and no visible drift, indicating good convergence of the model chains.

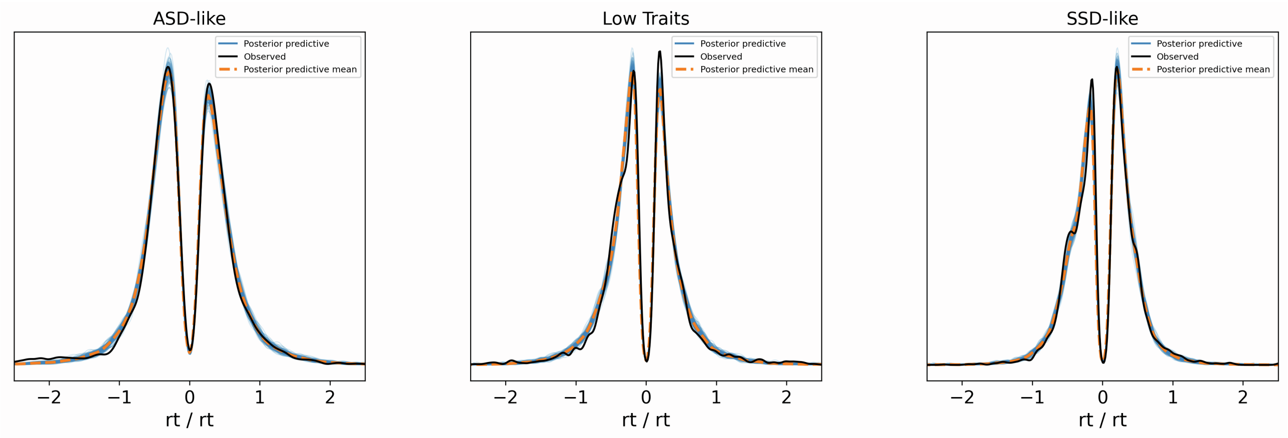

Figure S3. Group-level posterior predictive check (PPC) for the HDDM (Hierarchical Drift Diffusion Model), across groups. Each panel shows the observed reaction time (RT) distribution for each group (black line), overlaid with posterior predictive samples (blue shaded area) and the posterior predictive mean (orange dashed line) generated from the model (left panel: ASD-like; middle panel: Low Traits; right panel: SSD-like). The close alignment between the observed data and the posterior predictive distributions indicates that the model accurately captures the RT distributions across all three groups. Both the shape and central tendencies of the distributions are well recovered, suggesting a good overall model fit. RT values are plotted as negative for causal responses and positive for non-causal responses.

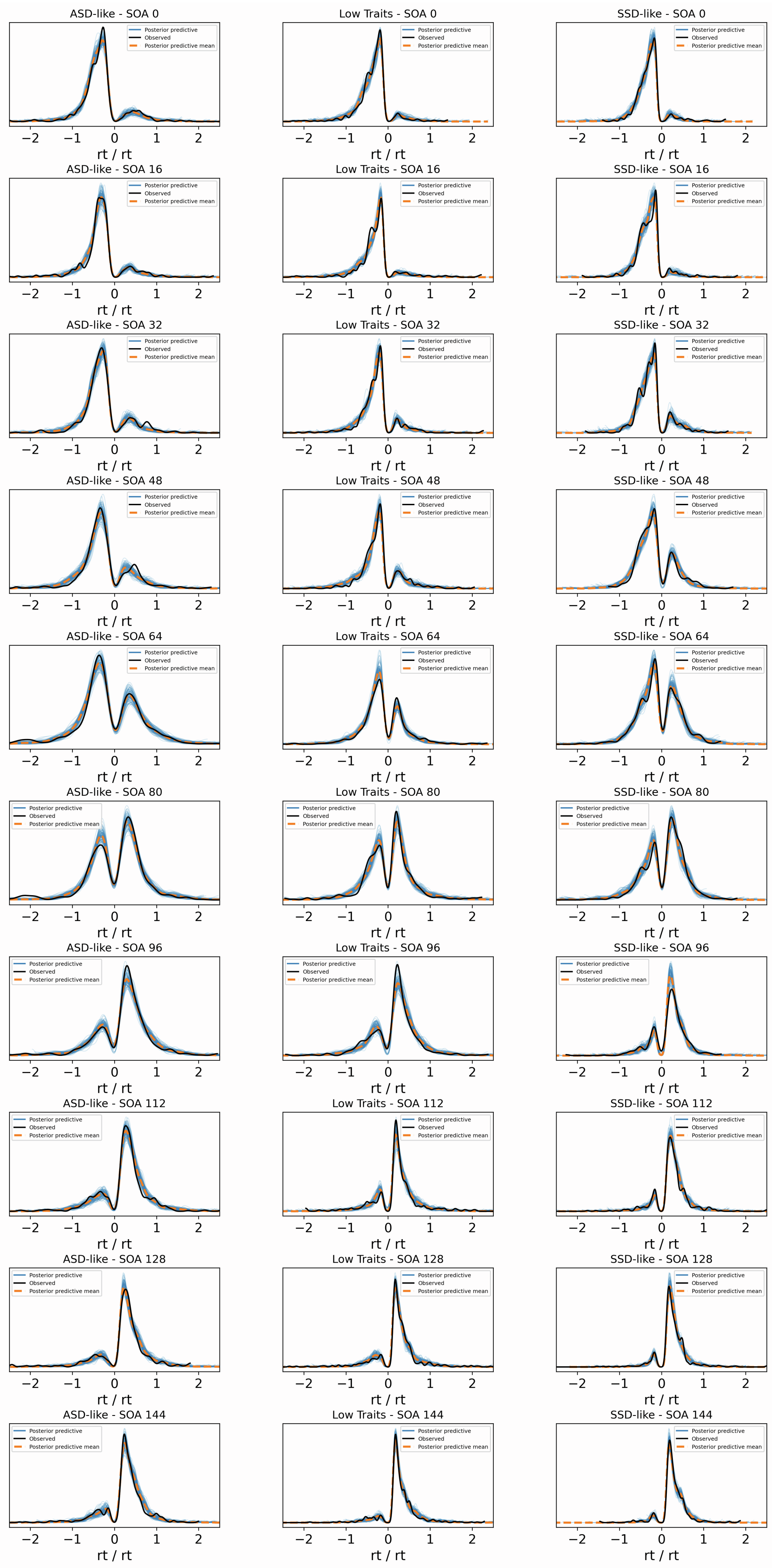

ASD-like - SOA 0
Low Traits - SOA 0
SSD-like - SOA 0
ASD-like - SOA 16
Low Traits - SOA 16
SSD-like - SOA 16
ASD-like - SOA 32
Low Traits - SOA 32
SSD-like - SOA 32
ASD-like - SOA 48
Low Traits - SOA 48
SSD-like - SOA 48
ASD-like - SOA 64
Low Traits - SOA 64
SSD-like - SOA 64
ASD-like - SOA 80
Low Traits - SOA 80
SSD-like - SOA 80
ASD-like - SOA 96
Low Traits - SOA 96
SSD-like - SOA 96
ASD-like - SOA 112
Low Traits - SOA 112
SSD-like - SOA 112
ASD-like - SOA 128
Low Traits - SOA 128
SSD-like - SOA 128
ASD-like - SOA 144
Low Traits - SOA 144
SSD-like - SOA 144
Posterior predictive
Observed
Posterior predictive mean
−2
−1
0
1
2
rt / rt

Figure S4. Posterior predictive check (PPC) for the HDDM (Hierarchical Drift Diffusion Model) across clusters and collision time lags. Each subplot shows the observed reaction time (RT) distribution for a specific participant cluster (ASD-like, Low Traits, or SSD-like) at a given collision time lag (black line), together with the posterior predictive mean (orange dashed line) and simulated posterior predictive distributions (blue shaded area) generated from the fitted HDDM. The close correspondence between observed and simulated RT distributions demonstrates that the model accurately captures the shape, variance, and central tendencies of the empirical data across conditions. RT values are plotted as negative for causal responses and positive for non-causal responses.

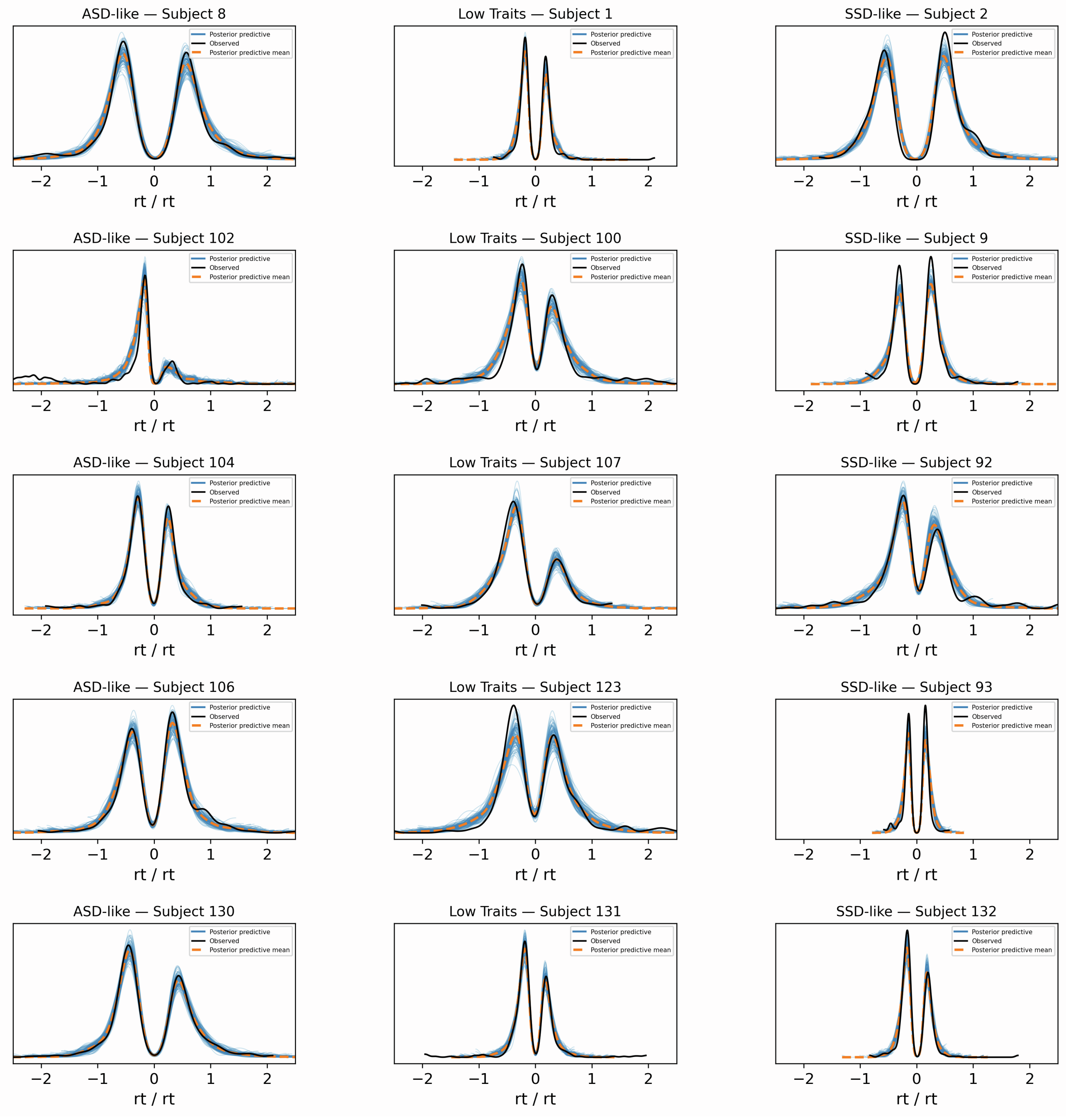

Figure S5. Individual-level Posterior predictive check (PPC) for the HDDM (Hierarchical Drift Diffusion Model). Posterior predictive checks at the individual level for randomly selected participants from the ASD-like, Low Traits, and SSD-like groups. Each subplot displays the observed reaction time (RT) distribution for a single participant (black line), the corresponding posterior predictive mean (orange dashed line), and the

posterior predictive distribution samples (blue shaded area) simulated from the fitted HDDM. Across individuals, the observed data are consistently well captured by the model-generated predictions. The HDDM successfully reproduces both the overall shape and variance of the empirical RT distributions. The tight overlap across participants confirms that the model not only fits group-level trends but also robustly accounts for individual variability in decision-making dynamics. RT values are plotted as negative for causal responses and positive for non-causal responses.

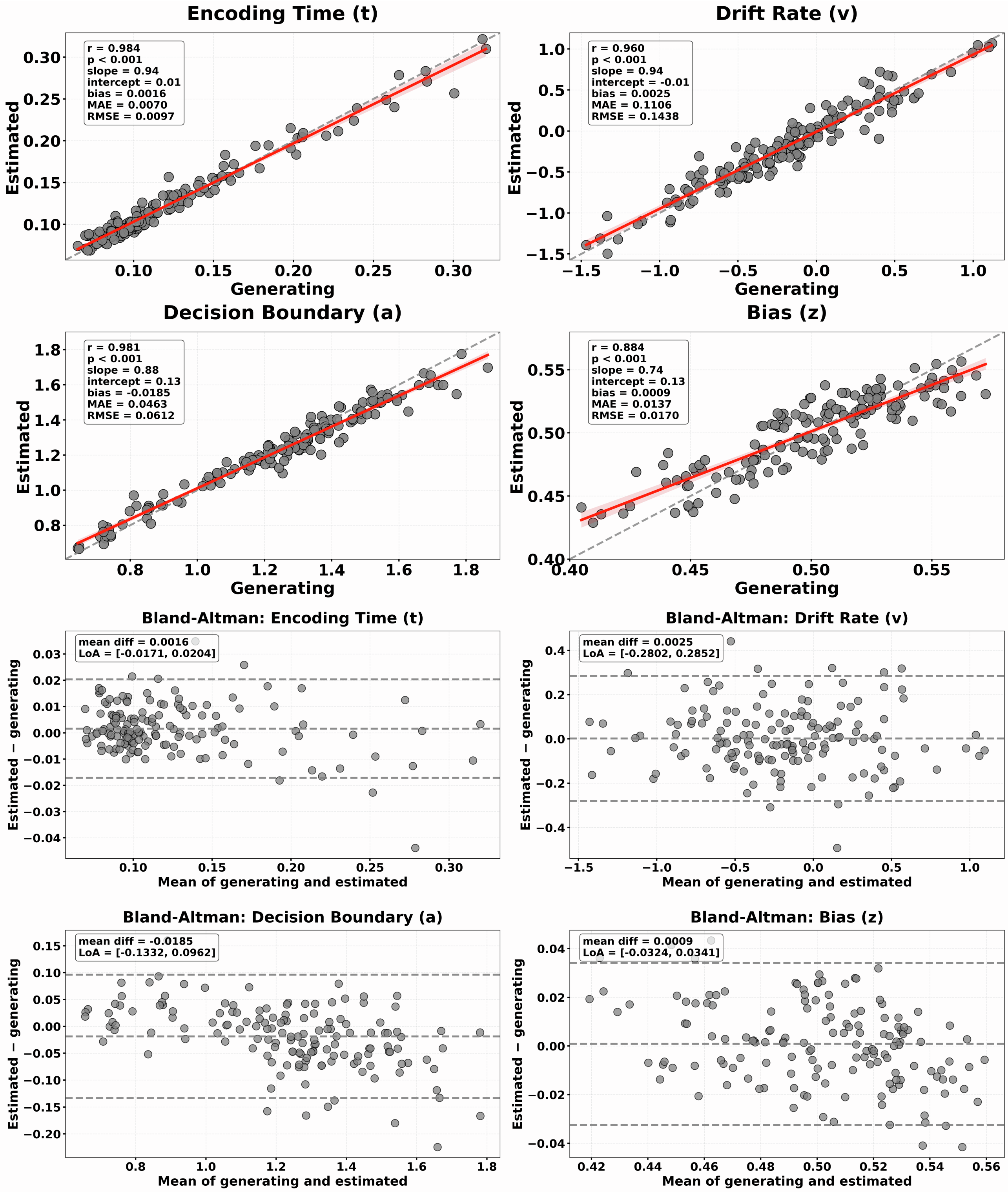

Figure S6. Parameter recovery analysis for the best-fitting HDDM. To assess the robustness and identifiability of the model estimates, we generated synthetic datasets by sampling subject-level parameters

from the posterior distributions of the best-fitting model and re-fitted these datasets using the same modeling procedure. Top panels show parameter recovery by plotting the generating (true) parameter values (x-axis) against the re-estimated values recovered from the simulated data (y-axis). Grey dashed lines indicate the identity line (y = x), and red lines show the regression fit. Strong and significant correlations ($p < 0.01$) across all parameters demonstrate reliable recovery of the underlying decision parameters, confirming that the observed effects are not artifacts of the estimation procedure. Recovery was strong for encoding time (t: r = 0.984, slope = 0.94, $R^2 = 0.969$), drift rate (v: r = 0.960, slope = 0.94, $R^2 = 0.922$), and boundary separation (a: r = 0.981, slope = 0.88, $R^2 = 0.963$). Starting-point bias (z) also showed reliable rank-order recovery (r = 0.884, $R^2 = 0.782$), with a reduced slope (0.74) reflecting attenuation toward intermediate values rather than directional bias; consistent with this, mean signed error and absolute error metrics were small.

Bottom panels present Bland–Altman plots (estimated − generating vs. mean of estimated and generating) to assess systematic over- or underestimation. Mean differences were close to zero across all parameters, confirming minimal directional bias; for z, differences varied with magnitude in a manner consistent with regression-to-the-mean effects rather than systematic misestimation.

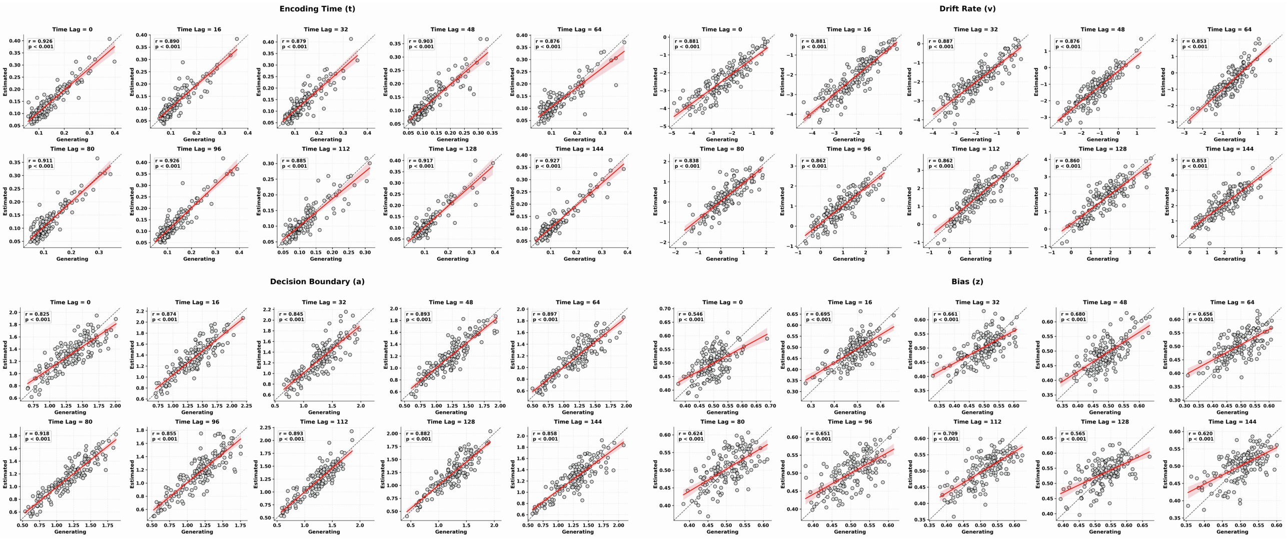

Figure S7. Parameter recovery analysis by time lag for the best-fitting HDDM. As an extension of the recovery analysis shown in Figure S6, here we assessed the identifiability of each model parameter separately at each collision time lag. For each parameter and time lag, synthetic datasets were generated from the posterior distributions of the best-fitting model and re-fitted using the same estimation procedure. The scatter plots display the correlations between generating (true) parameter values (x-axis) and re-estimated values (y-axis). Significant correlations ($p < 0.01$) across time lags demonstrate that the model reliably recovers the underlying decision parameters over different temporal contexts, further supporting the robustness of the parameter estimates.

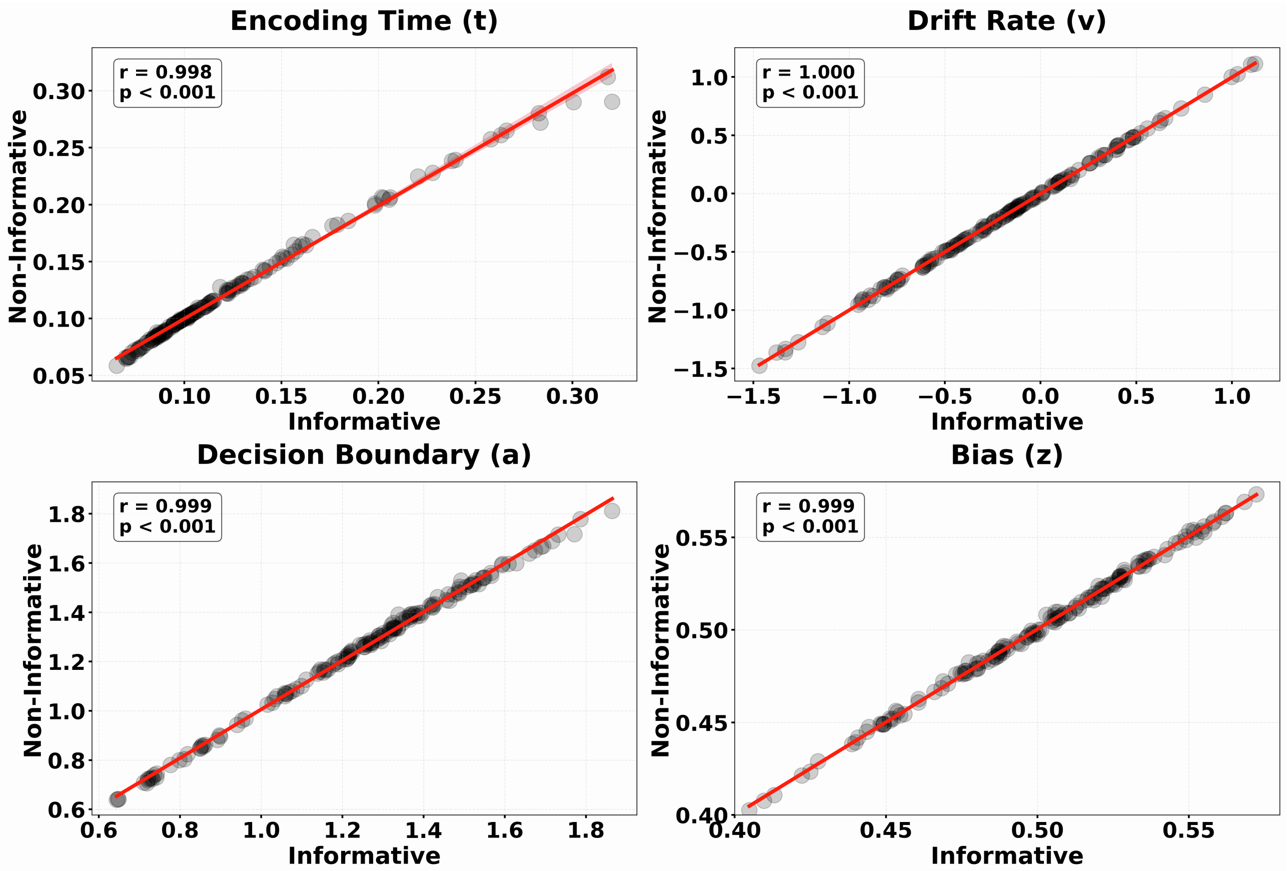

Figure S8. Robustness analysis of parameter estimates under different prior specifications. To assess the influence of prior assumptions on parameter estimation, the best-fitting HDDM was re-estimated using non-informative priors, which place minimal constraints on the parameter space and allow posterior estimates to be driven primarily by the data. The scatter plots show correlations between parameter estimates obtained with informative priors (x-axis) and those estimated with non-informative priors (y-axis). Strong and significant correlations for all parameters ($p < 0.001$) demonstrate that the model's parameter estimates are robust to prior specification and remain reliably identifiable across different prior settings.

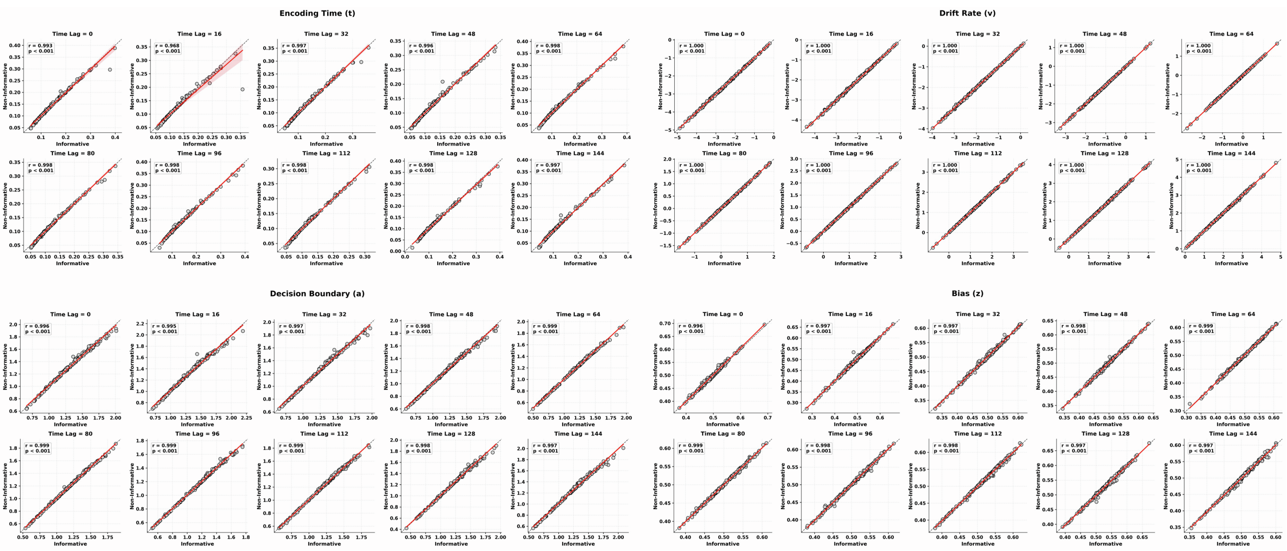

Figure S9. Robustness analysis of prior specification across time lags. To further evaluate the stability of parameter estimation, we repeated the prior sensitivity analysis separately for each parameter and collision time lag. Scatter plots show the correlations between parameter estimates obtained with informative priors (x-axis) and those estimated with non-informative priors (y-axis) for each time lag. Significant correlations across

all parameters and SOAs ($p < 0.001$) confirm that the parameter estimates are robust to prior specification and remain reliably identifiable across experimental conditions.

HDDMs	DIC	$\hat{R}$ max
1: No parameters depend on lag or cluster	38418	1.17
2: *t* depends on time lag	36192	1.04
3: *z* depends on time lag	27714	1.08
4: v depends on time lag	11116	1.09
5: *a* depends on time lag	38488	1.02
6: *t* depends on cluster	38417	1.13
7: z depends on cluster	38426	1.23
8: *v* depends on cluster	38410	1.36
9: *a* depends on cluster	38418	1.02
10: All parameters depend on time lag	9208	1.05
11: *z, v, a* depend on cluster; *t* depends on lag	36176	1.07
12: *z* and *v* depend on both lag and cluster; *t* depends on lag	9305	1.02
13: *v* and *a* depend on both lag and cluster; *t* depends on lag	9462	1.67
14: *z* and *a* depend on both lag and cluster; *t* depends on lag	22654	1.38
15: *z, v, a* depend on both lag and cluster (*t* fixed)	10746	1.02
16: *z, v, a* depend on lag and cluster; *t* depends on lag	9174	1.02
17: *z, v, a* depend on lag and cluster; *t* depends on cluster	10749	1.02
18: All parameters depend on both factors	9176	1.04

Table S1. Hierarchical drift–diffusion model (HDDM) comparisons. Model comparison results for all hierarchical drift–diffusion models (HDDMs) tested. For each model, the table reports the deviance information criterion (DIC) as an index of model fit (lower values indicate a better fit) and the maximum Gelman–Rubin convergence value ($\hat{R}$ max) as a convergence diagnostic (values < 1.1 indicate satisfactory MCMC convergence; see also STAR Methods). Among all candidate models, Model 16, in which the starting bias (z), drift rate (v), and decision boundary (a) depended on both collision time lag and participant cluster while encoding time (t) varied as a function of time lag, provided the best balance of model fit and convergence, as indicated by the lowest DIC and acceptable $\hat{R}$ values.
